# New DoS Defense Method Based on Strong Designated Verifier Signatures

**DOI:** 10.3390/s18092813

**Published:** 2018-08-26

**Authors:** Marcone Pereira de Almeida, Rafael Timóteo de Sousa Júnior, Luis Javier García Villalba, Tai-Hoon Kim

**Affiliations:** 1 Cyber Security INCT, University of Brasília, Brasília 70910-900, Brazil; marcone.almeida@redes.unb.br (M.P.d.A.); desousa@unb.br (R.T.d.S.J.); 2 Group of Analysis, Security and Systems (GASS), Department of Software Engineering and Artificial Intelligence (DISIA), Faculty of Computer Science and Engineering, Office 431, Universidad Complutense de Madrid (UCM), Calle Profesor José García Santesmases, 9, Ciudad Universitaria, 28040 Madrid, Spain; 3 Department of Convergence Security, Sungshin Women’s University, 249-1 Dongseon-Dong 3-ga, Seoul 136-742, Korea; taihoonn@daum.net

**Keywords:** Denial of Service (DoS) attacks, packet level authentication, traffic identification, digital signatures, designated verifier signatures

## Abstract

We present a novel technique for source authentication of a packet stream in a network, which intends to give guarantees that a specific network flow really comes from a claimed origin. This mechanism, named packet level authentication (PLA), can be an essential tool for addressing Denial of Service (DoS) attacks. Based on designated verifier signature schemes, our proposal is an appropriate and unprecedented solution applying digital signatures for DoS prevention. Our scheme does not rely on an expensive public-key infrastructure and makes use of light cryptography machinery that is suitable in the context of the Internet of Things (IoT). We analyze our proposed scheme as a defense measure considering known DoS attacks and present a formal proof of its resilience face to eventual adversaries. Furthermore, we compare our solution to already existent strategies, highlighting its advantages and drawbacks.

## 1. Introduction

The recent dissemination of Internet-based technologies such as peer-to-peer computing, cloud computing, and the Internet of Things yield to application scenarios where a non-measurable number of devices are connected and participate in multiple information flows supported by the Internet Protocol (IP) packet routing infrastructure. These new scenarios have introduced security challenges that are distinct mainly because they establish conditions to adversarial attacks in dimensions that are hardly manageable, especially in the case of denial of service attacks (DoS).

Recent distributed denial of services attacks have involved a massive number of IoT devices. These threats were among the causes of the Mirai botnet attacks [1], which used IoT devices infected by a malware that launched DoS attacks pushing massive volume traffic never seen before. Devices in the IoT ecosystem lack several security resources and, for that reason, they have been taken to trigger DoS attacks [2]. Considering the ever-increasing ubiquity and the persistent limitations of a large variety of IoT devices, regarding energy, memory, computing and communications capacity, it is a challenge to counter network security threats originated in these devices [3].

This paper presents a novel defense mechanism, consisting of a cryptographic protocol for the authentication of packet streams, which can be used to integrate a set of measures against DoS attacks. Our proposal makes use of basic cryptography tools, and to our best knowledge presents a new contribution in the way the digital signature is applied. Besides the description of this contribution, we show how our proposal is resilient to known DoS attacks, showing the advantages of its application in the security of IoT environments, mainly regarding DoS attacks inside IoT systems, but also that can be useful for countering attacks started from IoT devices to attain targets outside the IoT realm.

The remainder of this paper is organized as follows. Section 2 discusses related work highlighting some of the already known solutions to the DoS problem. In Section 3, we give an overview of cryptographic tools to be used in our work, and, in Section 4, we present the main result, by describing the proposed scheme. In Section 5, we present possible utilization scenarios where our scheme can be applied. In Section 6, we discuss how our proposal is resistant to DoS attacks and give a formal demonstration of it in Section 7. This paper is closed with our conclusion and future work goals in Section 8.

## 2. The Problem and Related Work

In this section, we introduce the problem focused in our proposal, presenting a brief overview of how DoS attacks can be carried out and discussing some important existing anti-DoS solutions, pointing out their weak aspects and highlighting where our proposal fits better than these existing ones.

### 2.1. The Adversarial Model

In the formalization of a security model, an important point is the description of the adversary. Some critical aspects of this description include how this entity can work to carry out threats to a target system.

The model considered in this work comprises a context where the parties are active actors in the network, i.e., they own computer processing and storage capacity and the capacity to transmit and receive through the network. We frequently refer to packets in the context of a packet-switching network, so our solution can be thought as one naturally adequate to IP networks. Furthermore, the proposal here can be extended to other network architectures, although we do not discuss such extensions. Another point is that we describe a security solution applied to the network level in the protocol stack, integrating our mechanism in the packets, but our security mechanism can also be extended to the link or the transport layer.

When a sender wants to give to the receiver the assurance that the traffic the receiver is getting is really sent as claimed, the sender will want to be identified and make sure the receiver can believe in the sender address informed in the arriving data traffic. These requirements guide the design of our proposed protocol which constitutes an authentication mechanism that aims to be useful for the considered scenario and will allow a sender to be identified by a receiver.

The adversary considered in this work will be carrying out attacks to subvert the authentication protocol executed between senders and receivers. To realize this goal, the adversary will use known DoS strategies, such as flooding, spoofing, content forgery, reflection/amplification, eavesdropping, replay and deny of capability attacks. The particular aspects of these threats concerning the IoT scenario are discussed in recent surveys such as [4,5].

### 2.2. DoS Attacks in the IoT Scenario

There is a broad set of vulnerabilities in IoT environments, compared to the ones in conventional computer environments [2]. First, IoT devices are highly heterogeneous with respect to communication protocols. They are also more prone to present mobility and usually run in an environment where different parties can share them (with shared or default passwords) and are also susceptible to be physically unprotected. In addition, IoT devices lack reliable authentication schemes although they generally provide network access through insecure services such as telnet and the very common unencrypted HTTP, constituting open doors for malware infection, as the recent cited case of the Mirai botnet attacks [1].

Another concern is related the fact that, given the nature of IoT devices and their ubiquity, most device owners are not used to procedures of firmware updates or bug corrections, differently from those enforced by modern operating systems in a conventional computer context. Moreover, when users are allowed to configure devices passwords, they tend to choose the easiest to memorize and frequently the shortest permitted one. Consequently, there is a higher success probability for adversaries stealing credentials mainly when these adversaries use password guessing tools, as discussed in [6,7]. Current IoT devices are plagued by this vulnerability consisting of a great number of device passwords that are weakly configured both by the manufacturing process and the unobservant end user. This vulnerability is the open door that is exploited in the Mirai botnet DDoS attack [8]. Hence, defenses against DoS attacks originated from IoT devices cannot rely on a change of behavior from the owners of these devices, despite the good practices openly publicized by the security community, such as the US-CERT alert concerning the Mirai botnet [9].

IoT devices lack elementary security resources, due mainly to their physical and computational limitations. This deficiency motivates the quest for security measures that suit the limited configuration of these devices without compromising the security requirements.

In this context, DoS attacks using IoT devices as reflectors is an important issue, mainly due to the vast number of devices in use nowadays, whihc is worrying because reflector attacks have been accounted as one of the most frequent forms of DoS attacks recently [3]. DoS attacks using these devices is a network security problem that imposes the same difficulties faced in the mitigation of DoS attacks in conventional computer system [10], but with the additional security challenges particular to IoT devices.

A previous work related to this paper [11] argues that the protection of an IoT network involves not only the IoT devices, but requires distributed solutions integrating security resources of IoT devices, IoT middleware, and IoT applications, which are entities commonly present in various IoT architectures. Thus, the security depends on end-to-end communications between these entities, highlighting the importance of mutually protecting communications in an IoT environment to keep it the most reliable. Furthermore, the IoT environment presents other network security demands in a context in which devices can communicate with each other. Thus, devising methods for discovering, identifying, and monitoring IoT devices is also critical [2].

By consider this situation and its security requirements, it is worth pointing out the importance of an authentication protocol that can be a distributed component of a comprehensive protection structure for IoT network instances. This observation motivates the defense method proposed in this paper.

### 2.3. Denial of Service Countermeasure Techniques

There are many DoS defense schemes proposed in the literature, using several paradigms, including machine learning, filtering schemes, information theory calculations, and cryptography. Recent reviews of DoS defense mechanisms can be found in [12,13,14].

In addition, there are efficient mechanisms for network activity tracking that have been the focus of several research works [15,16,17,18,19] and some of these results have been used as important components in anti-DoS solutions [20]. The focus on this aspect is due mainly to the fact that IP spoofing is the main vulnerability that is exploited by adversaries to launch DoS attacks. The possibility of tracing back [21] the source network addresses of an attacker is an important tool that not only supports anti-DoS measures but can also take a role in network authentication [22]. Packet inspection is a required basic technique to support and complement learning, filtering, classification and calculations used in the other techniques [23].

#### 2.3.1. Packet Filtering

An important paradigm for countering DoS is ingress-egress filtering which works mainly by enforcing some rule-based analysis of the source and destination addresses [20]. Some recent works make use of machine learning tools for modeling incoming and outgoing traffic to establish what is a normal or abnormal traffic pattern and to carry on packet filtering considering the modeled knowledge. Other filters take a statistic approach and build schemes based on looking up at the history of known traffic [24] where the known sources are mapped to their traffic pattern, a process that can help when an attack is going on. Some other DoS countermeasures are based on registering the route of traffic from frequent sources and making statistics of them [25], then considering the respective route fingerprint to filter eventual attacks.

The effectiveness of the schemes based in filtering depends on the power of the adversary, in the sense that a large-scale geographically spread attack can bypass any filtering policy, causing it to fail by producing unfair traffic blocking. Another aspect is that many solutions of this class demand cooperation among a large percentage of routers on the Internet to be effective. Performing this technique at the destination of the traffic does not result in effective protection.

#### 2.3.2. Cryptography, IPSec and VPN

The use of the IPSec protocol [18] could be a solution to the presented security challenges, but the deployment of IPSec in large-scale still represents a considerable price regarding computational complexity, which makes it an inefficient approach. Furthermore, the problem of DoS attacks described in this paper demands a form of IPSec configuration which not just applies encryption, but also needs authentication or integrity mechanisms [26], because the lack of an authentication/integrity check mechanism opens the door for attacks on IPSec [26,27]. Bellare and Namprempre [28] discussed the necessity of authentication beyond the mere use of cryptography, pointing out the essential analysis that must be done to set some IPSec configurations. Thus, when IPSec is considered as a solution to the challenges posed by DoS attacks, it not merely a question of using cryptography but also of choosing appropriate authentication schemes. Consequently, care must be taken when cryptography is applied since there is the risk of the solution resulting in other challenges.

Furthermore, in the adversarial scenario that we are considering in this work, the access to a public key infrastructure (e.g., access to digital certificates and validation chain verification) can itself bring some vulnerabilities. The point is that in this kind of context a third party, the certificate authority (CA), takes an important role and the access to it can leak information that the adversary can use to perform attacks, such as man-in-the-middle and impersonating the CA.

#### 2.3.3. Bloom Filter Forwarding

Originally, Bloom filter was defined in 1970 by Burton Howard Bloom in [29], where he proposed a space-efficient data structure that allows element addition and a probabilistic set membership test. Among some useful properties of this data structure, it can be shown that it is information theoretically infeasible to revert a Bloom filter to the inputs that resulted in a given state, and at the same time it permits membership tests in linear constant time complexity [30]. The structure is probabilistically checkable [29] in the sense that there is a non-zero probability of *false positives*, that is, there exists a chance for the filter to answer positively about a not included element. Therefore, the probability of *false negatives* is null, i.e., when the search for an element returns negative, there is no chance of error. A concern is that the probability of false positives grows with the number of items added to the filter [31]. However, a bigger Bloom filter will support a lower false positive rate. Furthermore, it is not easy to modify the data structure to allow element deletion operations, although some proposals can be found in the literature [32,33] that allow removing elements from the filter.

A Bloom filter can be implemented as an array of length *n* bits, such that a set of *k* (cryptographic) hash functions $\left\{{hash}_{0},\;{hash}_{1}\ldots \;{hash}_{k}\right\}$ can be used to map an element to *k* indexes in the array. An item inclusion is committed by applying all the *k* hash functions and joining all the corresponding outputted bits set to 1, for each of the *k* hash functions. When used under some privacy, e.g., doing it by cryptographic hashes, it is hard for an attacker to add some element in the data structure in a way that it counts as a correct element member of the set, since the set of hash function is kept in privacy.

The Bloom filter has been used as a supporting tool for routing monitoring, multicast address management, and DoS control since it allows the receiver to check if a flow passed through some specific path. However, the scheme has been shown to be vulnerable to several types of attacks [30]. As mentioned, it is hard to manage element deletion with Bloom filters, and this fact is pointed as an important defense issue. Adversary collusion can use packet forgery or spoofing in the network as the main tools to perform DoS attacks.

The work in [34] presents an interesting analysis of the trade-off between the dimension of the information that is cataloged and the size of the Bloom filter. The authors show that the size of the Bloom filter can be a concern when considering the nature of the managed information: a sub-dimensioned Bloom filter can raise false positive rates, and an over-dimensioned one consumes processing and networking capacities. When a Bloom filter is a component of a network security solution, care must be taken for keeping the size of the Bloom filter appropriately adjustable according to the nature of the network traffic, considering that a DoS attack can abruptly change the characteristics of this traffic.

#### 2.3.4. Cryptographic Puzzles

Another classic cryptographic scheme is the one based on cryptographic puzzles, introduced by Merkle [35]. The central idea in client puzzles is to provide a way for a client to prove his legitimacy by solving a challenging problem sent by the server (moderately hard, but not intractable function), an operation that demands some computational effort from the client and so denominated *proof of work*. The use of cryptographic puzzles as a tool for resource usage control appeared in [36], and then inspired other cryptographic countermeasures for DoS attacks [37,38,39,40,41]. Puzzles are used to force a sender to spend some time solving a difficult task, so that this sender earns credits that allow it to transmit. The receiver will demand more of these credits when the sender wants to continue to transmit.

In the access control scheme in [36], the server challenges the client demanding him to reverse some cryptographic hash function or some cryptographic signature. In the case of hash functions, the server asks the client to perform computation until he finds a value *x* such that the chosen hash function applied to this value corresponds to an output value with a chosen suffix, for instance, $hash\left(x\right)=\left[\ldots 001101\right]$. The central idea here is of preimage resistance, i.e., the computational difficulty to find a value *x* such that $hash\left(x\right)=y$ since this hardness is a fundamental property of any secure hash function and therefore some probabilistic time interval is needed in reversing their output [37].

The work in [37] introduced DoS defense mechanisms based in cryptographic puzzles. There, the authors proposed a defense for protocols like TCP and SSL against connection depletion DoS attacks. However, in that work, the client puzzle runs as an application-level program in the computer client, a solution that is itself a problem. Oppositely, Feng et al. [41,42] argued about the necessity of placing the puzzles-based DoS defenses in the network and transport layers. They highlight the weakness of solutions that put client puzzle functions as application programs, resulting in undefended lower layer protocols such as IP and TCP. In fact, they point out the necessity of deploying puzzle mechanisms inside the IP/TCP architecture in order to be effective against DoS attacks, considering that threats to applications are a concern whose treatment cannot be restrained to application-level solutions.

### 2.4. DoS Defenses in IoT Scenarios

There are in the literature several proposals for DoS detection mechanisms for IoT. Recent surveys that exhaustively list the most recent works in this area are [4,43,44]. These works, in general, propose a monitoring approach that does not effectively stop an attack, just alarming of their occurrence. Up to our knowledge, there are few proposals that present means to control attacks in IoT networks after attack detection.

In [45], the authors present an analysis of the attacks based in telnet access to several devices and propose a defense mechanism comprising a honeypot and a sandbox, which are used to attract the adversary and to analyze its behavior. This work considers security from the sole viewpoint of the devices and does not find the IoT environment as a source of attacks.

Another proposal comprises a firewall which is used to provide security for the communications within an IoT environment [46]. In this strategy, rules are applied to filter the access to services and resources of the environment, and also to filter which devices can communicate to the outside Internet.

Brachmann et al. [10] proposes to use cryptography to provide security in communications between IoT devices, setting up an end-to-end protection mechanism. The authors presented an adversarial model, listing the threats that can be covered by their solution. The authors of [47] proposed another inter-device secure scheme, using cryptographic hash algorithms to provide mutual authentication between devices. The authors in [48] suggested the use of certificate-based authentication mechanisms to provide mutual authentication among end users of a wireless sensor network. Salmon et al. [49] proposes a collaborative mechanism to provide an intrusion detection system that warns the network about attacks, considering the network devices passively monitoring DoS attacks.

### 2.5. Contribution and Overview

In this paper, we propose a defense mechanism comprising the authentication of network packet flows employing strong designated verifier signatures. Our scheme can be joined with a DoS detection scheme, where it could take a role in a proactive side of DoS defense, doing it in a capability-filtering approach. The detection side is needed to warn in the case of an attack, which cannot be done just by capability-filtering scheme [50,51]. We argue that our proposed scheme is new because it makes use of cryptographic tools in an original way that brings the following benefits:

- Deployment in network and transport layers: Our solution can be fit in IP packets or in TCP segments. It can also be used as an authentication/identification tool in other protocols or services that establish a network flow.
- Agility: Current solutions are based on heavy cryptographic machinery in routers [15,16,37,38,39,40] or assign to the routers some filtering task with high computing overhead per packet [19,20,21,22,24]. Our proposal can provide on demand validation of a packet stream with very low computing complexity.
- Our proposal does not require modifying the network infrastructure: Apart from most known solutions from the literature, our scheme does not change the IP or TCP architecture.
- Low communications complexity: Our solution does not overburden the communications, and our protocol does not require any interaction among the parties and certification authority servers. In addition, it does not rely on key exchange protocols that demand a previous setup of confidential channels between parties. Our proposal adds merely a short signature in a fraction of the transmitted packets, a quantity defined as a security parameter, and therefore does not interfere in the traffic load.
- Accurate traceback to the sender: The proposed mechanism allows the identification of the sender, an attribute that can be used by the receiver to throw out malicious packet streams. When integrated to other security measures, this feature helps to detect ongoing attacks, allowing the receiver and other network elements to be alerted when a DoS attack is in course.
- Resilient to the interference of active adversaries: We give a formal proof of the resilience of our identification mechanism, showing how much resistant is the protocol face a malicious party controlling the network, and that can interfere in the messages used to establish the proposed scheme.

## 3. Cryptographic Tools and Assumptions

This section presents an overview of some cryptographic tools used in the construction of our scheme. We discuss the concept of digital signatures and how it is related to authentication and give an overview of the proof paradigms used for demonstrating the security of these protocols.

### 3.1. Digital Signature and Message Authentication

A digital signature is a primitive that allows a signer to convince a verifier that a statement is valid utilizing a digital proof. A similar concept is of *message authentication*, both methods allowing validation of data content, i.e., they permit to verify that a given document, information, was approved by a certain entity [52].

A digital signature must be universal in the sense that everyone can check the validity of what is being proved. A fundamental requirement from a digital signature method is that it must be unforgeable [52]:

- A signer can efficiently create a signature of any document, and everyone can efficiently check the validity of a given signature.
- It is not feasible to forge a signature in the name of another user, that is, one cannot create a signature to a document that the other did not sign.

On the other hand, authentication methods consider an insecure channel and are only verifiable by communicating parties which created the authentication tag. It is expected that authentication systems satisfy the following requirements over an insecure channel [52]:

- Each of the communicating parties can efficiently create an authenticating tag to any message of its choice.
- Each communicating party can efficiently verify an authentication tag of a given message.
- It is infeasible to any external party (not in the communicating set) to create an authenticating tag.

More formally, a signature scheme is defined as follows:

- A key generation algorithm G: the algorithm $\left(K_{S},K_{P}\right)\leftarrow G\left(1^{K}\right)$ produces the respective secret and public keys, and it considers the security parameter *k*, where $1^{k}$ is the unary representation of the intended security level. G is probabilistic in the sense that it considers some random value ω to process the keys generation.
- The signature algorithm Σ: The signer, by owning both secret and public keys, can execute algorithm Σ to produce the signature σ of a given message *m*. The signature algorithm can be probabilistic, making use of a random value as an input.
- The verification algorithm V: The verifier takes the signature σ and the public key $K_{P}$ and checks if σ is a valid signature of the message *m*.

**Figure 1:**
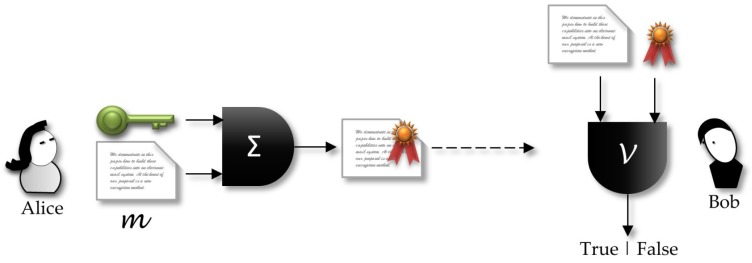
Signature of a message *m* by the sender, and the verification process by the receiver.

Figure 1 shows a sender, Alice, who holds a private key $K_{P}$. Using a signature algorithm Σ, she inputs a *clear-text* and the secret key. The output is the correspondent (*text, signature*) pair that will be sent to the receiver. Any receiver that gets the signed message can verify the signature using the sender public key.

Working with digital signatures standards that use public key based digital certificates, such as the ones in the X.509 family, public and secret keys will be under the management of a third-party entity, the certification authority (CA). Thus, a more realistic scenario should include this third entity in Figure 1.

### 3.2. Proof of Security Paradigms

As we already mentioned in Section 2, a proof of security of a protocol requires a precise and rigorous definition. Therefore, a formal definition of a secure signature demands an adequate formalization of what it must consider as the adversarial model, and what kind of proof it will use. In the next subsections, we briefly discuss some of the main paradigms of proof of security for signature schemes.

#### 3.2.1. Simulation-Based Formulation

An important security paradigm is the concept of *semantic security* [53]. This proof of security compares what the adversary can learn by inspecting the ciphertext (i.e., the cryptosystem output) and what he can learn by himself without looking at the ciphertext. The argument is that, if what the adversary can learn observing the ciphertext is not more than what he can learn doing a simulation himself [54], the information the cryptosystem leak will not help him to break the scheme. The proof holds if the amount of information learned by looking at the ciphertext is very close to what is captured by inspecting the adversary simulated by himself.

The paradigm of simulation holds that the security of protocols can be proved by stating that everything the adversary can learn is limited to what he can simulate by himself. In other words, the fact that he can inspect encrypted messages does not give him any advantage that could not be already learned before the encrypted output was created.

**Figure 2:**
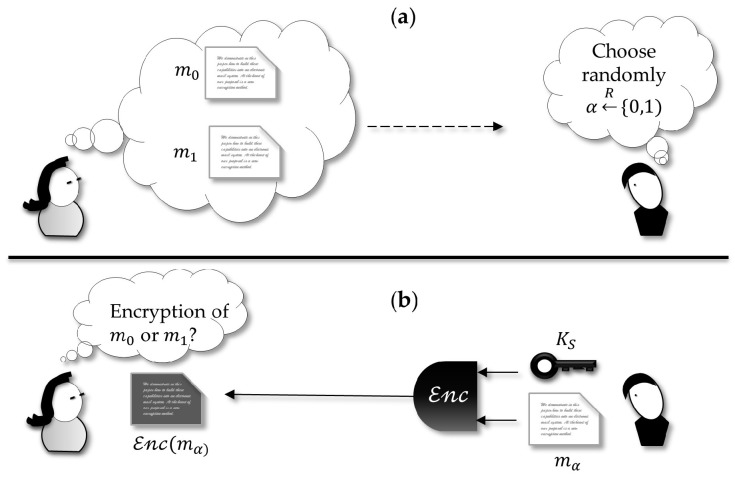
(**a**) The challenger Alice takes two messages randomly chosen and sends them to the signer Bob. (**b**) The signer flips a coin and chooses either 0 or 1, then he encrypts one of the messages and sends the enciphered message back to the challenger, who wins the game if she finds which *m* was encrypted.

This is commonly illustrated by a hypothetical scenario where an adversary challenges an honest party that is executing an encryption scheme (Figure 2). The adversary chooses two messages that he gets randomly and submits them to the honest party, demanding this party as an encryption oracle. The honest encipher flips a coin and so chooses randomly one of the two messages, encrypts it and sends it back to the challenger adversary. In this case, the probability of attack against the cryptosystem is put in terms of the chance the adversary wins the following game: if he can guess with an advantage no greater than 0.5 (not better than a random choice) which one is the clear-text corresponding to the enciphered, then the ciphertext leaks no information useful for breaking the cryptosystem. In other words, the encrypted message is indistinguishable from a random message.

#### 3.2.2. Proof by Reduction

Another approach for the proof of security gives us a mathematical measure of how feasible it is for an adversary to break the secure protocol by showing that the underlying hard problem that sustains this protocol must be broken by the adversary to attack the protocol successfully. This kind of proof construction is named *proof by reduction*. This paradigm is largely used in the literature. For instance, when a secure protocol is based in the assumption of the hardness of the discrete logarithm problem, the proof occurs by showing that any attack strategy to the protocol can be used for constructing a mechanism to solve the discrete logarithms.

#### 3.2.3. Random Oracle Model

Another widely used proof method is the so-called *random oracle* model due to Bellare and Rogaway [55]. It considers an idealized uniformly random function that is accessed by the protocol participants only in a black-box way, i.e., the random function can be called for evaluations, but no description of it is given. By its turn, when called two or more times under the same argument, the random oracle returns the same answer. Then, the security is proved considering this idealized functionality, i.e., recognizing the existence of functionality that has a stronger security assumption than whatever can be computationally run to concretize it. There is no real computing system which can run as a true random oracle [56]; in practice, random oracles are substituted by hash functions, as these functions are considered an acceptable oracle candidate. That distance between what is idealized and what can be really done computationally is a source of debate about the model [56].

Therefore, the random oracle model takes a vital role in the proof of secure digital signature schemes [57], mainly because hash functions are a fundamental building block in digital signature protocols.

### 3.3. Secure Digital Signature Definitions

The seminal work of Pointcheval and Stern [57,58,59] was the first to argue about the security of signature schemes, and their work was settled under the random oracle methodology. It considered an adversarial model in which the adversaries know a list of message–signature pairs and then try to break the protocol by analyzing these signed messages. Thus, the referred work gives a formal adversarial model for signature protocols, pointing the main attacks against signature systems, such as when the adversary can disclose the signer secret key, breaking the entire system.

Among the important attacks modeled, the cited work underlines the adversary ability to forge the signatures of messages, which is then named *universal forgery*. Another described attack comprises the ability to provide a new message–signature pair for a random message, i.e., the adversary is not looking at a specific signature for a given message. This attack is named *existential forgery*. Given these definitions, the mentioned work proposes the following description of security for digital signature schemes:

***Secure Signature Scheme** [57]: A signature scheme is secure if an existential forgery is computationally impossible, even under an adaptively chosen message attack.*

Existential unforgeability under a chosen message attack for a digital signature scheme (G,Σ,V) as pointed in definition is defined using the following game between a challenger and an adversary A [60]:

- *Setup*: The challenger calls the key-generation algorithm G and gets keys pair $K_{S}$ and $K_{P}$; the adversary A receives the public key $K_{P}$.
- *Queries*: Adversary A chooses *n* messages of his choice $m_{0},\ldots ,m_{n}$ and requests the challenger the correspondent signatures $\left\{\sigma_{0},\ldots ,\sigma_{n}\right\}$, where $\sigma_{i}=\Sigma {(K}_{S},m_{i})$.
- Output: Adversary A wins the game when he eventually outputs a pair (M,σ), where M was not asked in the previous step.

### 3.4. Undeniable Signature

As an extension to the original design, Chaum, and Antwerpen [60] proposed the idea of *undeniable signature*, in which the verifier needs the cooperation of the signer to verify the signature effectively. Thus, apart from the classical signatures, an undeniable signature cannot be verified openly by everyone. This scheme can be more realistic for many applications because traditional digital signatures can be copied and tested by everyone anytime while undeniable signature allows the signer to manage who can check the signature or can later refuse to cooperate in the validation interaction.

**Figure 3:**
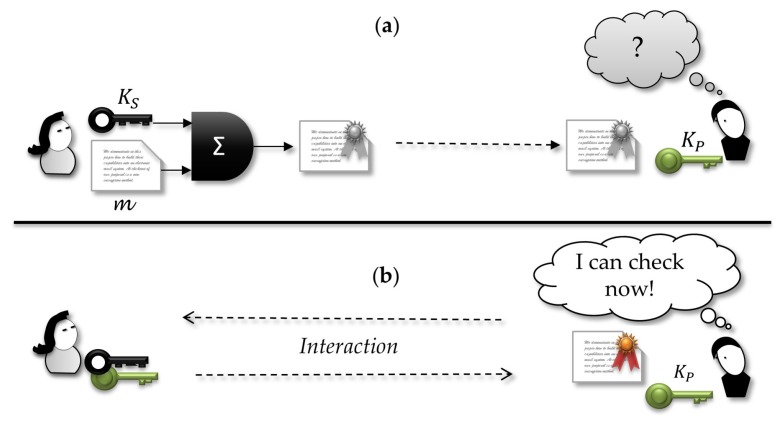
In undeniable signature, (**a**) the receiver is not able to verify the validity of a signature until (**b**) the signer agrees to perform a validation interaction.

Figure 3 illustrates undeniable signature. First, the signer sends a valid signed document to the verifier, although they do not have yet any interaction for verification. Thus, in the first moment, the signature is indistinguishable from a random bit sequence. When they agree on an interaction, all signature information can be checked by the receiver and only after that it can make sense to him.

Originally, the authors intended to give the signer the control of who and when a signed document is checkable, an operation that requires an interaction with the signer. However, the signer does not hold full control of who can verify the signature, neither has control over how many times it can be done. In this sense, the protocol does not reach its intended goal of full management of who can verify the authenticity of a signature. Effectively, the signer controls only the moment of verification. Furthermore, one disadvantage of this scheme is the complexity of the interaction needed for verification, which can make the protocol useless for some applications.

### 3.5. Designated Verifier Signature—DVS

Jakobsson, Sako and Impagliazzo [61] introduced the concept of designated verifier signatures (DVS). Extending the notion of undeniable signature, DVS allows the signer to choose which verifiers can check the signature validity; furthermore, the verification procedure does not demand interaction with the signer. Thus, this kind of signature presents the advantage of operating in a disconnected way, which is appropriate for the problem managed in the present paper.

Different from traditional signature schemes, DVS adds the restriction that if Bob tries to forward the signature to a third party, Cindy, she will not be convinced about who really signed the document, because it could be a production of Bob. In other words, the receiver Bob can forge a dummy signature by himself, such that this new one looks indistinguishable from the original created by Alice. This property is an interesting characteristic, non-transferability, that thwarts Bob’s intent of leaking a signature in a message, just because only Bob can really verify and believe the signature. More formally, we have the following definition:

***Designated verifier signature** [61]: Let $\left(P_{A},P_{B}\right)$ be a protocol for Alice to prove the truth of the statement θ to Bob. We say that Bob is a designated verifier if the following is true: for any protocol $\left(P_{A},P_{B}^{\prime },P_{C}\right)$ involving Alice, Bob, and Cindy, in which Bob proves the truth of ϑ to Cindy, there is another protocol $\left(P_{B}^{\prime \prime },P_{C}\right)$ such that Bob can perform the calculations of $P_{B}^{\prime \prime }$, and Cindy cannot distinguish transcripts of $\left(P_{A},P_{B}^{\prime },P_{C}\right)$ from those of $\left(P_{B}^{\prime \prime },P_{C}\right)$.*

**Figure 4:**
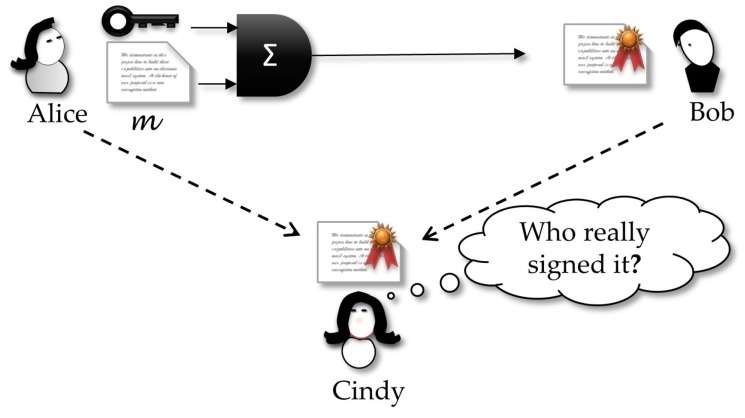
Bob is able to verify the validity of the signature as the designated verifier. The view of Cindy is that the signatures are indistinguishable of any other created by Bob.

The above definition can be interpreted by following game (Figure 4): anything a curious Cindy can capture from Bob does not help her to believe it is the real proof sent by Alice or is a dummy proof produced by Bob. In other words, if Bob sends Cindy the transcription of the protocol by which he really received the signature from Alice, Cindy cannot distinguish this from the transcription of a protocol by which she received the claimed signature from Bob. This way, Cindy does not have any reason for believing Bob.

### 3.6. Strong Designated Verifier Signature —SDVS

**Figure 5:**
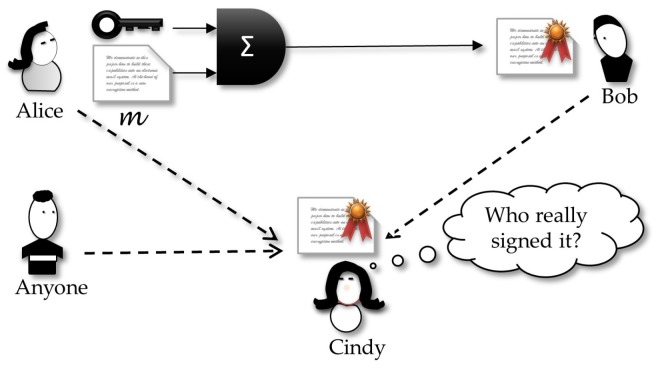
In SDVS, a curious Cindy cannot decide who is the author of the signature, because this one is indistinguishable from any other that anybody could create.

The concept of *strong designated verifier* signatures protocols extends the original DVS and makes it run securely even in the context where it is widely believed that Bob is honest and will not engage in a forgery and forward the resulting forged signature as a genuine one. In this case, even if a cheating Alice leaks a signature she addressed to Bob, then Cindy can get it but will not check if this a valid signature, because only Bob can do this verification. This impossibility is since not only Bob can forge a signature that looks indistinguishable from the original created by Alice, but everyone else can do the same. That property is the illustrated in Figure 5 showing that this new scheme works in a scenario where not only Bob can leak the signature received from Alice, but also Alice can be dishonest and try to forward a signature created by her and sent to Bob. In the two cases, a curious Cindy will not be sure about who really created the signature.

***Strong designated verifier signature** [62]: Let P(A,B) be a protocol for Alice to prove the truth of the statement *Ω* to Bob. We say that P(A,B) is a strong designated verifier proof if anybody can produce identically distributed transcripts that are indistinguishable from those of P(A,B), for everybody, except for Bob.*

This definition requires that the transcript of the protocol execution between Alice and Bob does not leak any clue about the real proof, since the transcript is indistinguishable from any other created by any person, including Alice, Bob or whoever else. This property fits appropriately to our proposal in the present paper.

In this case, a curious Cindy will equivocally interpret the signature origin. In our proposal, we use this in the following convenient way: Alice must sign a contract and send it to Bob, but she knows that they are using an insecure channel in which a curious and malicious Cindy can eavesdrop the signed message. Thus, Cindy will be sure about the origin of the document, but the SDVS property implies that she will not be able to convince any other, except Bob, about the authorship of the signature. However, if Alice is worried about Cindy’s curiosity, Alice can get the message she wants to sign and mix it with *n* other different messages. Now, Alice will sign all these n+1 messages but be producing a valid signature only relative to the original message. At the other end of the channel, Bob will be able to check all signed message and discern the correct message and its signature. The convenient property of SDVS is that now Cindy will not realize which message–signature pair is the valid one, because all the wiretapped pairs look like the same.

## 4. Description of Our Scheme

In this section, we integrate the concepts described in preceding sections to present our proposed protocol, showing how those cryptographic tools are used for constructing an authentication method suitable for a packet switching network, such as the IP routing infrastructure.

### 4.1. Tagging Packets

Our basic conception is a protocol that allows a receiver of a packet stream be sure about the authenticity, or identification, of an alleged source in the network. As already pointed out, this protocol uses designated verifier signature as its basic cryptographic tool.

The idea of inserting a tag in network packets enabling some capability-based control is not new. For example, some works have proposed the insertion of tags (containing, e.g., a hash of some data flow property) in packets and schemes based on this form of capability mechanism already exist [63,64]. In [63], the authors inserted a Bloom filter calculated from packet addresses, thus constructing a control for the IP forwarding mechanism that has inspired other DoS defense measures [64].

A naive scheme would be one in which the sender signs all the packets and attaches the signature to the IP header. In this case, classical signature schemes are not enough protective, because an adversary can easily spoof all the traffic and replay it. Thus, the signature machinery will not help in any aspect. Moreover, the price to produce signatures for every packet is high, as is the price of checking every signature in the receiver. We propose a more efficient way to use signatures. Furthermore, we consider that an anti-DoS cryptography-based mechanism must employ some protective measure regarding packets that have already been sent, for the case of the adversary trying to replay network traffic, and this can be a very expensive task. A more efficient management of this task is another contribution of our protocol.

**Figure 6:**
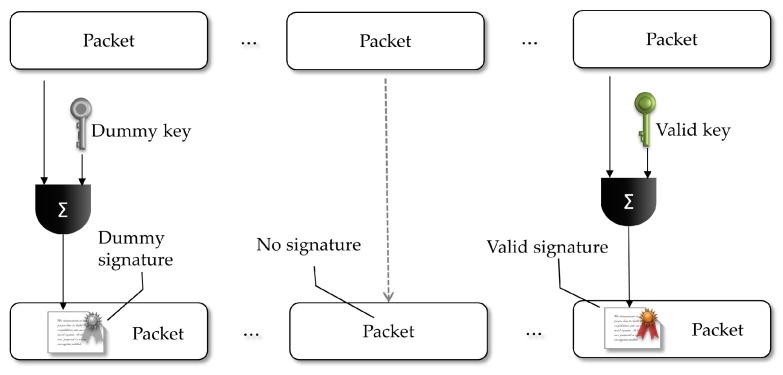
The proposed mode of generating packets signatures by the sender, thus producing packets with dummy and coherent signatures and packets with no signature.

Our strategy explores the equivocation property of SDVS schemes, allowing the sender to sign some packets in a valid way (recognizable by the designated receiver) and to insert dummy signatures in some other packets. More specifically, we put the sender to use his private key and the receiver public key in an SDVS protocol to sign some packets coherently. The sender also generates dummy signatures using chosen random keys. As shown in Section 3, it is indistinguishable to the adversary which signatures are coherent and which are the dummy. However, the (designated) receiver will be able to distinguish if any of the packets was coherently signed by the sender and will keep track of that authentication signal in memory. As shown in Figure 6, with the goal of sustaining a chain of authentication signals, the sender will always choose randomly what specific packet he will sign coherently, doing it periodically for the forwarded groups of packets. The complete scheme defines two sets of packets: (1) packets with signatures, being those coherent or dummy ones; and (2) packets with no signatures.

### 4.2. Design Details and Parameters of the Proposed Scheme

This section presents our protocol details, showing its parameters and how they fit in our complete solution. The proposed scheme uses the following security parameters:

- *n*: the size of the sequence of packets to be considered when a set of signatures must be inserted;
- Σ: the total number of packets in which some signature will be inserted, i.e., for each *n* packets sequence, Σ have a signature (valid or dummy), n−Σ have no one;
- σ: the total number of valid signatures in a set of Σ signatures inserted in an *n*-packet segment; and
- τ: the threshold number of *n*-packet segments without valid signatures, i.e., the receiver will expect at most τ·n received packets with no valid signature. Otherwise, the authentication status is switched off for that sender.

The proposed protocol works as follows. The sender, to prove he is an authentic source of a network packet stream will exchange information needed to get the destination public key (we do not suppose this public key must be accessed from a public key infrastructure; the supposition here is that the receiver just keeps it publicly accessible).

Once this setup phase is concluded, for every *n* packet to be transmitted, the sender will execute one round of the algorithm, that is: randomly choose Σ packets where some signature will be inserted, and among these choose σ of them which will get valid signatures. In its turn, the receiver will be able to check the validity of the signatures and count the valid ones, thus being aware to verify if the threshold of τ rounds is reached with no valid signature received, interpreting this as a loss of authentication status for the sender.

In a formal systematic specification, we describe our protocol as the sequence of the following steps:

- **Initialization**: This phase starts with the parties openly agreeing about the security parameter $1^{N}$ and then calculating their respective secret and public keys using the key generation algorithm G. The parties make their public keys openly accessible. That is described in the Algorithm 1.
**Algorithm 1:** Initialization **begin**  Alice gets ${(K}_{P}^{A},K_{S}^{A})\overset{R}{\leftarrow }G\left(1^{N}\right)$  Bob gets ${(K}_{P}^{B},K_{S}^{B})\overset{R}{\leftarrow }G\left(1^{N}\right)$ **end**
- **Signature**: After that initial phase, the signer Alice, for a round that must be executed for every *n* packets to be transmitted, must randomly choose the subset Σ among them, the ones in which she will fit some signature. In addition, inside this subset, she must at random choose the σ packets that will receive valid designated signature that can effectively be verified by the receiver Bob. The code in Algorithm 2 describes this signature process.
**Algorithm 2:** Alice (sender/signer).
 **begin**  **for**
$\left\{p_{1},\ldots ,p_{n}\right\}$
**do**   Generates dummy keys: ${(K}_{Dummy}^{A},K_{Dummy}^{A})\overset{R}{\leftarrow }G\left(1^{N}\right)$   Randomly chooses: $\Sigma =\{p_{1},\ldots ,p_{\left|\Sigma \right|}\}$   Randomly chooses: σ⊂Σ   **for**
$p_{i}\subset \sigma$
**do**    $\sigma_{i}\leftarrow Sign\left(p_{i},K_{S}^{A},K_{P}^{B}\right)$    Append($p_{i}|\sigma_{i}$)   **end**   **for**
$p_{i}\subset \Sigma \setminus \sigma$
**do**
    ${\;\sigma }_{j}\;\leftarrow Sign\left(p_{j},K_{dummy}^{A},K_{dummy}^{B}\right)$    Append($p_{i}|\sigma_{i}$)   **end**  **end** **end**
- **Signature verification**: For each signed packet, Bob must verify if the inserted signature is valid and counts to find at least one valid signature in every sequence of τ·n received packets. thus, for Bob, if the source of the packet is not able to put a valid designated packet in such intervals, it is assumed that with a good probability (we will give a formal description of this in Section 6) the source is not authentic, or someone is trying to cheat. All this process is as the code in Algorithm 3.
**Algorithm 3:** Bob (receiver/verifier).
 **begin**  **for**
*every received signed packet: $\left(p_{i}|\sigma_{i}\right)$*
**do**
   **if**
*$Verify(\sigma_{i})$ is True*
**then**
    update the threshold authentication (τ)   **end**   **else**    update the threshold authentication (τ)   **end**  **end** **end**
- **Simulator**: Similar to proofs in SDVS schemes in [61,62], here a simulator takes an important role in the proof of security of the protocol. In the security proof of the SDVS, the existence a simulator that allows mimicking the transcript of an authentic conversation is used as an argument of the hardness to anyone, except the designated verifier, to distinguish what is a valid designated signature and a random one. The simulator will drop some coins to get some randomness, take as inputs the public keys $\left(K_{P}^{A},K_{P}^{B}\right)$ and the packet to be signed, and uses a simulator Simul of the underlying SDVS (because as the SDVS is supposed to be secure, it is required that a simulator exists for it) and with some scheme will return a signature σ that by the correspondent properties will give guarantees that the returned signature is indistinguishable from the valid one, except for the designated verifier. The simulator heuristic is described in Algorithm 4.
**Algorithm 4:** Simulator. **Input**: *n* packets: $\Sigma^{\prime }=\left\{p_{1},\ldots ,p_{n}\right\};$
  $K_{Dummy}^{A},K_{Dummy}^{B}\leftarrow G\left(1^{N}\right)$ **begin**  **for**
$p_{i}^{\prime }\;in\;\Sigma^{\prime }$
**do**   $\sigma_{i}\;\leftarrow Simul\left(p_{i}^{{}^{\prime }},K_{Dummy}^{A},K_{Dummy}^{B}\right)$  **end** **end**

We argue that, if some adversary holds any strategy to efficiently distinguish between the set of signed packets took from the simulator described above and a set indeed signed by Alice, such adversary strategy can be used to construct an efficient distinguisher for an underlying SDVS, contradicting the supposed properties of the scheme, i.e., that is infeasible for anyone, except the designated verifier, to recognize a valid signature when challenged with a pair containing a simulated and a valid signature. In other words, the ability of an adversary to distinguish the simulator output implies the ability to distinguish the used SDVS output.

We also argue that, if there is a simulator such as the one described above, that can be used by anybody to simulate an indistinguishable signature, one can synthesize a sequence of signed packets that is indistinguishable of that really signed by Alice. Therefore, if any strategy exists that can give some advantage in recognizing the one coming out of this simulator and the one really signed by Alice, this strategy can break the designated property of the underlying SDVS scheme, by distinguishing a pair of messages.

It must be considered that there are two types of signatures coming from Alice: the valid ones and the others created using randomness, which we designate $\sigma_{v}^{a}$ and $\sigma_{r}^{a}$, respectively. They both are indistinguishable from the ones synthesized by the simulator, called here $\sigma^{Sim}$. Therefore, what someone needs to distinguish between two sets of elements is at least to be able to recognize some individuals in each of the sets. That is, any strategy able to distinguish the two sets must recognize at least one signature that was created by Alice in one set and another signature output by the simulator in the other side. However, by the SDVS properties, the pairs $\sigma_{v}^{a},\sigma^{Sim}$ and $\sigma_{r}^{a},\sigma^{Sim}$ both have elements that are indistinguishable, and these two types of tuples are the ones the adversary strategy must distinct.

Thus, if one strategy can make a distinction between two sequences as described above, we can use this strategy to construct a distinguisher that violates the privacy property of the SDVS scheme by recognizing a valid signature. This can be done by simply creating two sequences of messages, generating keys and signing like the simulator signs, and then randomly choosing one of these sequences and randomly choosing where to open a slot to insert the signed message we want to verify as valid or not. Then, we submit this construction to the adversary strategy that must distinguish the entire sequence and eventually point the one with a valid signature when this is truly the case. This way we have shown that if there is any advantage given by the adversary strategy in recognizing the entire sequence of signed messages, it will give us an advantage in breaking the SDVS scheme, which is a contradiction to the supposition that it is secure.

## 5. Envisaged Application Scenarios

We present two possible utilization scenarios in which our solution works as an element of a DoS defense mechanism. The first scenario is designed as a packet authentication resource for a general purpose network, while the second one is intended to IoT environments.

### 5.1. Application in a General Network Scenario

We present a possible utilization scenario in which our solution works as a DoS defense mechanism. It considers DoS threats over an Internet-connected network where IoT devices are supported. The proposed strategy, in this case, is that the defense scheme is activated just when a DoS attack is detected by a receiver, that is, under an offensive. Thus, while under this attack, the receiver will require the senders to only transmit signed traffic, since the receiver will allow only this traffic possibly being aided by routers and firewalls nearby.

**Figure 7:**
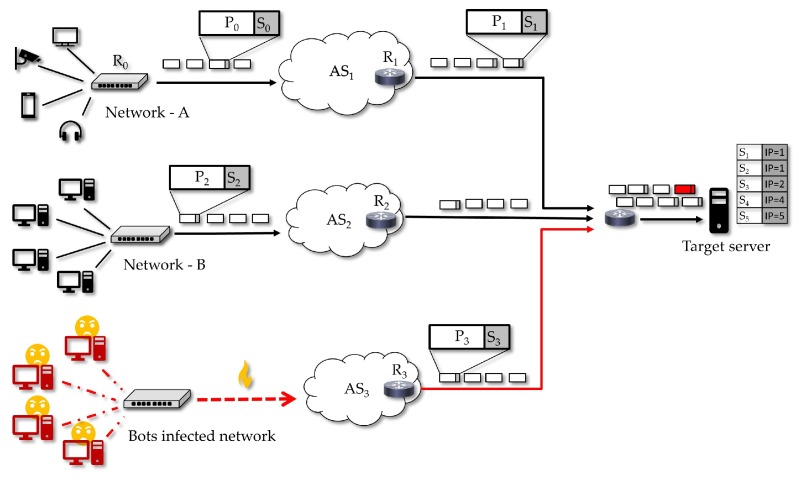
A possible utilization scenario for the proposed DoS defense. During a DoS attack, the target server requires signed traffic from the senders and, since in this case just *Network-A* and *Network-B* can do it, the other sender is blocked.

In the scenario illustrated in Figure 7, it is considered that a target server is facing a DoS attack from a botnet and that this attack can result in the server eventually denying access to legitimate clients in *Network-A* and *Network-B*. Using our scheme, when an attack is detected by the target server (or by an IDS that alarms this server), it can activate the defense scheme by requiring that every sender who wants to transmit must appropriately sign the sent traffic packets. By performing the required signature (an operation that can be performed by endpoint devices, access routers and autonomous systems (AS) routers), *Network-A* and *Network-B* will have their network traffic recognized by the target, while any unsolicited traffic can be blocked by the server, possibly with the help of nearby routers and firewalls.

**Figure 8:**
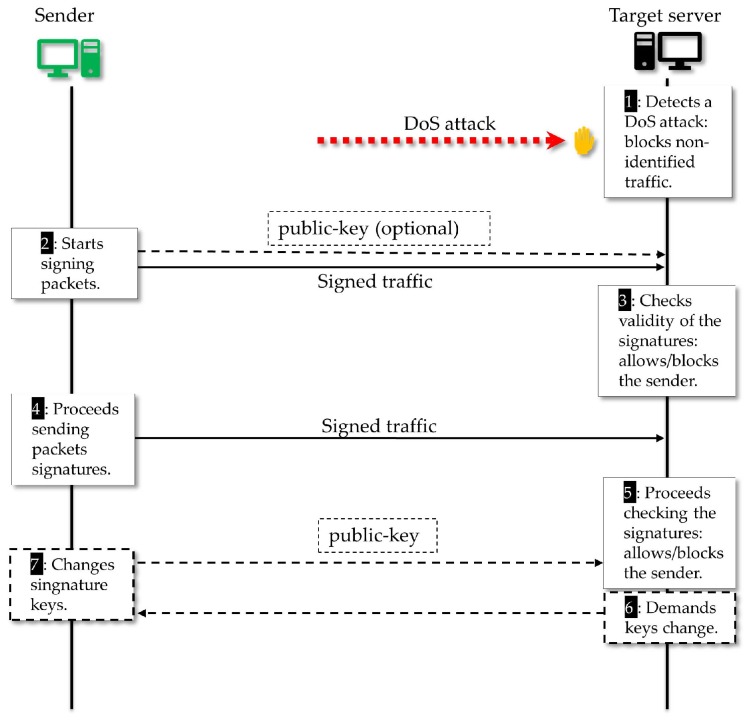
Overview of the proposed defense dynamics showing the interaction between sender and receiver.

In Figure 8, we present an overview of the proposed protocol dynamics. In Step (1), the target server detects a DoS attack and activates the defense mechanism. Given the requirement of the target server, in Step (2), the *Sender* starts the packet signature process. There are several ways the receiver can get the sender public-key: as long it is public, it is optional for the sender to forward it together with the traffic, we highlight that in this phase there is no interaction with a third party like a *certification authority* (CA), as in a conventional public-key based signatures. It is broadly known that the verification by a public CA *chain of trust* is needed when a conventional signature is used to check the validity concerning personal or organizational identity. We also highlight that the SDVS signature scheme used in our mechanism was created to protect the anonymity of the signer. Hence, we argue that our choice is enough for our objective which is to provide a way to distinguish a particular sender flow and decide to block or to allow this flow. Therefore, if a malicious adversary tries to impersonate a legitimate sender, e.g., by copying its public key, he will not succeed, unless he knows how to break the signature scheme. We emphasize that we have a light signature scheme that does not depend on a third-party and that is enough for the *identification* of a source, a feature that is useful for network traffic filtering, which is done by the target server in Step (5). After that, if the sender wants to continue sending traffic, it must keep signing the packets and, if the target server intends to stay receiving that specific network flow, it must carry on checking the sender signatures, as shown in Step (5).

It is interesting to give an example of how our proposed scheme is resistant to spoofing attacks, since they are an important tool for performing DoS attacks. Considering an adversary that is spoofing traffic in the name of a public entity such as Google.com, with the attacker for instance forging traffic as it was originated in one of this company DNS servers, for example, the one whose IP address is 8.8.8.8. This adversary, when trying to use this address to run a spoofing attack will be asked by a receiver about the public key linked to 8.8.8.8. The adversary will answer with a public key of his choice (even a key copied from the real server). The receiver does not have any option, but to believe that the answer is a legitimate key (i.e., a real Google.com key). The point here is that our scheme works perfectly if the adversary tries to send DoS traffic, just because the receiver that activates our scheme in this situation will be able to distinguish the adversary flow and block it, no matter the adversary signatures are not really a genuine proof of a Google signature. Hence, the DoS traffic is blocked.

We reiterate that our proposed defense allows attack filtering and blocking and that it does not open a door for spoofing attacks, a concern for other network defenses that can be dealt if these defenses are joined to ours. In fact, with the proposed scheme activated, even if an adversary decides to appropriately sign DoS traffic, its network flow will be distinguished and blocked.

We also highlight that one advantage of our proposed scheme that makes it suitable for simple IoT devices concerns the private key. First, this key is not required to be managed by a certification authority, but it could be, e.g., if the router of the IoT perimeter performs the function of the signer. It is is the same case for even the border router in an autonomous system (AS). Secondly, that private key differs from a password, in the sense that it does not have to be memorized by the users or device owners, so it could be created and updated by some hardware functionally of the device, and this operation can get some simple intervention from the user, such as simply pressing a button for a random time. Finally, the scheme does not demand this key to be strong, information theoretically speaking. The requirement is that the adversary must expend a time to break or extract the key, this period being enough to turn infeasible a massive a DoS attack.

As mentioned in Section 2.2, the vulnerability consequent to the easiness for an adversary to hack passwords and then take control of IoT devices is really an important challenge. Our proposal is to use authentication failures to trigger a filter, during the detection of an attack, thus blocking all the traffic except the traffic flows which can be identified. In our proposal, the receiver is able to block the senders that are not trusted and allow those that are identified as well behaved ones.

Our argument is that, if an active adversary tries to send DoS traffic, this adversary will be distinguished. Even if this adversary tries to cheat with a private-key (used to sign messages) chosen on its will, the related network flow will be distinguished. In addition, if an active adversary tries to cheat by periodically changing the private-key or using a private-key stolen from other devices, the receiver will continue to be aware of who is really sending harmless traffic. In the attack context, the receiver will be filtering traffic, and the allowed flows will be those recognized as coming from well-behaving sources.

Another interesting case is when the adversary hacks an already identified device thus getting its password to start sending malicious traffic in the name of a sender rated as well behaved. That was the case in the Mirai botnet DDoS attack [8]. In our proposal, such coordinated attacks can be managed if the receiver requires that well-rated senders change their private signature keys (Steps (6) and (7) in Figure 8). We argue that it is infeasible for the adversary to coordinate its attack operations in view of this change in the entire set of owned devices, since this a costly procedure both computationally and regarding communication efforts. The defense controller is able to pose an increasingly costly challenge to the attacker, even if the attacker has provable full control of the devices.

### 5.2. Application within an IoT Network Scenario

As the IoT concept can involve potentially different hardware platforms and operating systems, IoT middleware, communication protocols, data models, types of applications, and security policies, among other components, we refer not to a single, homogeneous and cohesive Internet of Things, but to the various incarnations or instances of the Internet of Things that support simultaneous and parallel IoT applications.

**Figure 9:**
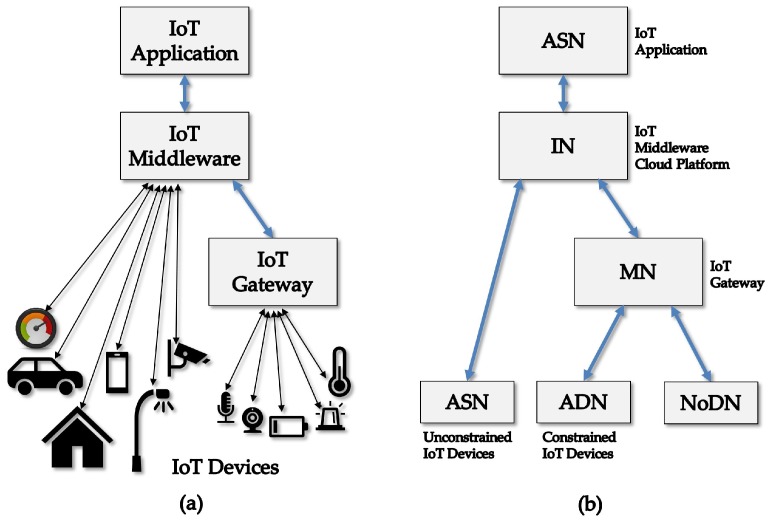
A common IoT Instance (**a**); and its corresponding oneM2M functional configuration (**b**).

A typical organization for IoT instances comprises IoT devices, both constrained (simple sensors and actuators) and relatively unconstrained ones (smart objects), IoT gateways that provide protocol conversions and mediation services for some of these devices, IoT middleware, and IoT applications, as shown in Figure 9a. The IoT middleware effectively manages the various heterogeneous components that must interconnect and interoperate. Specifically, the IoT middleware is a software layer between the physical layer of devices and their interfaces and the application layer that integrates the logic and control of the IoT instance. Because easy integration and communication among heterogeneous components are desired, the IoT middleware is required to provide a set of programming abstractions, including device discovery, access to services, and administration tasks.

In addition, in Figure 9b presents the mapping of the described common IoT instance to the functional architecture of oneM2M [65], which is one of the most supported bodies to promote standards for M2M and the Internet of Things. According to this figure, in oneM2M, IoT instances comprise infrastructure nodes (IN), middle nodes (MN), application service nodes (ASN), application dedicate nodes (ADN) and non-oneM2M nodes (NoDN). NoDNs correspond to simple sensors and actuators while ADNs are smart objects. ASNs can be smart objects or client application devices or gateways for simple devices. MNs are unconstrained devices. INs are cloud platforms or servers for the main middleware modules.

The security solution adopted in oneM2M [66] literally mandates that the functional entities need to be mutually identified and authenticated, to protect against unauthorized access and Denial of Service attacks. This mutual authentication enables to additionally provide encryption and integrity protection for the exchange of messages across the reference points that constitute the interfaces among oneM2M entities. Besides, the application entities that require similar protection for their own information exchanges can be provisioned with the same security method to their communications.

We adopt a point of view similar to the oneM2M standard, and other authors, that entity and message authentication are essential measures contributing to mitigating Denial of Service attacks in IoT. The authentication protocol proposed in the present paper suits the oneM2M standard specification and presents the advantageous feature of dynamically adapting the number of signed packets, thus reducing computation efforts compared to a protocol that mandates signing every packet. This feature is particularly interesting for constrained IoT devices.

However, we recognize that DoS attacks are rather complex distributed and combined operations such as scanning victims, performing intrusion, malware distribution, command and control, reporting, performing attacks on command, etc. [67]. These articulated operations cannot be countered just with cryptography and cryptographic protocols such as the authentication protocol proposed in this paper.

Regarding DoS attacks related to IoT, it is worth distinguishing DoS attacks internal to the IoT instance or the IoT realm from the attacks that use IoT devices as bots to attack other systems and services, such as the DNS, corporate web servers, cloud platforms, content providers, and so on.

In a previous related paper [11], we argue that, when the security requirements demand preventing DoS and DDoS attacks against IoT instances, a possible counter-measure is that the IoT middleware should count and correlate received messages with the objective to filter and to attend authentic requests while discarding messages classified as vehicles for DoS/DDoS.

However, this is not a general measure to counter other variants of DDoS, such as those originating in IoT devices against entities in other IoT instances or in other networks, systems, and services. These attacks seem to require fully distributed countermeasures which are IoT security measures that must not necessarily be implemented inside the middleware but just count on the middleware support to be deployed in the form of applications collaborating with devices in fully distributed operations. Thus, it is required that the devices implement local security services to be used for the distributed security applications. Using the same principles of a fully distributed model proposed for MANETs in [68], IoT instances can count on these fully distributed security measures that can be organized using the devices local security services cooperating with central security application hubs. For instance, a smart device can embed a local intrusion detection system (LIDS), a local anomaly detection module, a local autonomic trust reasoning module, a local DDoS probe, a local logbook, and so. These probes can announce their services throughout the IoT middleware to be used by the corresponding distributed IoT security application.

**Figure 10:**
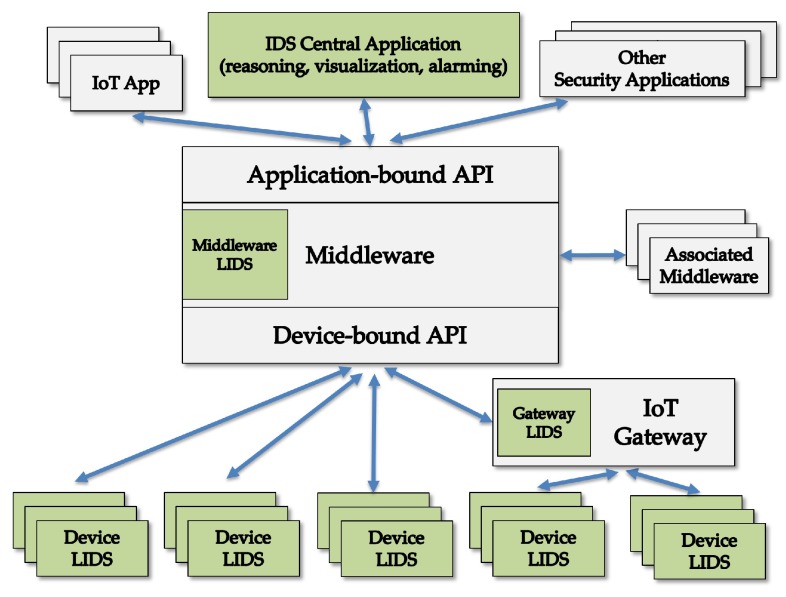
A fully distributed IDS for IoT.

Figure 10 exemplifies a distributed intrusion detection system (DIDS) for IoT in which a central application implements IDS reasoning, visualization and alarming, and this application collaborates with local intrusion detection systems (LIDS). These LIDS are located in smart devices and count on the IoT middleware itself to support the information flows. This DIDS is one possible companion measure to the authentication protocol proposed in this paper so that together they are viewed as an authentication-based filtering scheme against DoS.

Considering the attacks against passwords of IoT devices, as referred to in Section 2.2, the LIDS plays the role of detecting the misuse of the password in a device and then sending corresponding alarms to the other DIDS components. The rules applied by the LIDS, in this case, may include for instance verifications regarding old passwords never or rarely used, modifications of passwords, passwords being used to access device services with messages coming from sources that are outside the IoT realm, dictionary attacks against device passwords, and other similar ones.

## 6. Resilience of Our Scheme in Face of DoS Attacks

In this section, we formalize the measure of how much resilience our proposal brings against DoS attacks by showing what chances an adversary can earn by attacking a network in which the senders and receivers apply our proposed protocol. With this purpose in mind, we analyze common attack scenarios already pointed out in previous sections.

### 6.1. Spoofing Attacks

We discuss in this section how our strategy brings difficulties to who plan to perform a DoS attack based on spoofing. Any attempt to spoof traffic, e.g., by copying or forging packets with the intention to inject malicious traffic, will be successful if the attacker can synthesize a valid signature in the SDVS scheme. Therefore, we demonstrate that there is no efficient adversary strategy to inject forged traffic that brings him authentication with a likely probability unless the adversary breaks the underlying SDVS scheme.

As already mentioned, no one except the designated verifier will recognize a valid signed message. Thus, we argue that, unless the adversary knows how to break the privacy property of the SDVS scheme, the adversary cannot deal with the probability of σ/Σ when he tries to cheat in every stream of *n* packets. Thus, the strategy that remains for spoofing or replay attack is to copy all the signed packets and to replay them, an event that should be considered as an alert of DoS by the receiver. It is interesting also to note that in IP the insertion of packets is not always free for the attacker; however, we are not relying on any control mechanism such as packet sequencing, or any other congestion control.

Another point is that parameter τ is the threshold that puts the limits of how many groups of *n* packets the receiver will tolerate if not finding any valid signature among them. In fact, if the attacker has got a good guess of which ones are the packet with a valid signature, the mere copy of that packet for future replay in a spoofed packet stream will allow him to transmit no more than 2·τ·n packets (a sequence of τ·n packets with no valid signature, and another one with a forged signature) until the receiver be warned by a fault regarding the valid signature threshold. However, considering that this hypothetical guess is not feasible, the adversary will be allowed to inject a τ·n sequence of a malicious packet, i.e., if it is required a tight and small τ=1, the possibility is just of n malicious packets.

### 6.2. Flooding Attacks

The adversary can also add malicious packets to the regular network flow. By this, he will be drifting forward the authentic packets, but the chances of transparently doing it depends on the knowledge of the position of the valid signatures on the stream. Regardless, pushing spurious packets in the packet stream, the adversary can disturb the proposed authentication mechanism because this can turn a *n* packets segment in a non-authenticated one. However, the adversary must fit the inserted traffic in a convenient place. Remembering that the rate of packets with a valid signature is σ/Σ for every n packets, the expected distance between any two valid signatures is n/σ, i.e., the best strategy to the adversary is to put the malicious traffic on the half way between to valid signed packets, but he will do it just if he previously distinguished valid signatures from dummy ones. Therefore, he will not perform better than a procedure of random position inserting.

Therefore, the adversary cannot know where the valid signed packet is and will see every signed packet as the same, and the strategy that remains for him is for inserting malicious traffic between all signed packets. As the sender will be signing packets randomly, the space where malicious traffic can be inserted will be close to n/Σ in probability.

### 6.3. Denial of Capability Attacks

We have already defined capability in Section 2. When an adversary attempts to disturb an authentication mechanism based on capabilities, i.e., based on something the user owns for proving his identity (e.g., a key, a token, or a digital certificate), we call it a denial of capability attack [22]. Our scheme proposes a capability based on packet content, and an attempt of dropping packets in the network level must be considered as a denial of capability attack. Packet dropping has its proper countermeasures [69] in protocols such as IP, including congestion control and retransmission mechanisms. Notwithstanding, an adversary can launch a packet content manipulation as an effective tool to achieve denial of capabilities attacks.

Therefore, to effectively realize a denial of capability attack against our scheme, the adversary will need to recognize which packets are the ones with designated and valid signatures. As already mentioned, finding it demands breaking the SDVS scheme. If the adversary drops all (or a good portion of) the signed packets, or if he modifies the content of them, it will trouble the authentication mechanism.

Hence, the chances the adversary has for successfully denying an authentic sender and not being caught depends on the rate of signed packets Σ/n and on the rate of valid signed packets σ/Σ. The higher the rate Σ/n of signatures per packets, the more the adversary will get obfuscated concerning which packets he must target the dropping attack, and the least strategy is by manipulating all the Σ signed packets. An attack dropping all the packets is impossible to fix because there is no defense to such an event. Consequently, if the adversary cannot recognize valid signatures, he can only drop all signatures because of the resilience of the protocol faces the loss of signature packets.

### 6.4. Content Forgery Attack

The question here concerns the chances for the adversary that follows any strategy of manipulating a subset of signed packets. An attacker can attempt to thwart an authentication artifact by manipulating the content of the packet, by modifying or just dropping the signatures. First, if the adversary tries to throw out a targeted packet entirely, he will not reach his objective because the retransmission mechanisms will make the sender repeat the same packet on the network. Alternatively, by trying to modify δ of the Σ packets, the chance of success will raise with δ, but, for every time the sender attempts to keep the authentication alive, the adversary must play the same attack, until attaining the τ threshold. Doing so many manipulations on the packet content, the adversary will get exposed by his intention of offense. For example, if the attacker tries to frustrate an authentication stream *t* times, the chance of success will be ${\left(\delta /\Sigma \right)}^{t}$, and he will have to manipulate t·δ packets.

For a practical example, when we have a scheme set by signed Σ=10, σ=1 for each n=100 packets, with the tolerance of up τ=5, if the adversary tries to drop five of these signed packets, he will have a chance of 5/10=50% of being successful one of the times he tries to thwart the connection. However, the sender will insist on the connection by retransmitting signed packets, which leaves the adversary with the chance of ${\left(5/10\right)}^{5}≅0.0312$ after the fifth performance by the sender.

## 7. Finite State Analysis Regarding the Resilience of the Proposed Protocol

In this section, we present a proof of how secure our protocol is, showing how resilient it is in the face of a considered adversarial model. We proceed using an essential formal proof tool: finite state machines.

Protocol design demands proof of security, and formal specification is one of several methodologies for this task. Directly speaking, a formal specification is the use of mathematical techniques to give certainty that a protocol in analysis conforms to some precisely written notion. In this line of reasoning, the modeling of protocols by finite state machines, or automata, is a paradigm often used for computer language specification, and very common in network protocols specification and performance evaluation.

In the field of secure protocols design, several works make use of formal specification for finite state analysis [70,71,72], and in some of these works the use of automated verification is applied [73,74], especially when a manual proof is infeasible.

### 7.1. Finite State Automata

Briefly speaking, a finite state automaton (FSA), or finite state machine, is an abstraction of a step-by-ptep process. With this powerful tool, it is possible to model any computing process. The model gives an abstraction of binary relations on a set. The elements of the set are the states, and the relationships between these elements are called transitions. A transition S→Q is the one from the state *S* ruled by a correspondent condition that when satisfied makes the local state to be updated to *Q*. More formally:

***Finite state automaton*** [75]—a finite state automaton is a tuple $\left(Q,\Sigma ,q_{0},F,\delta \right)$ where *Q* is a non-empty set of states, among them $q_{0}$ is called the initial state. Σ is a non-empty finite alphabet, and F⊆Q is the subset of final/accepting states. The transition function is δ:Q×Σ→Q, that is, when applied to an element of alphabet return the next state: e.g., δ: ($q_{1},$‘*c*’) $\to \;q_{2}$.

**Figure 11:**
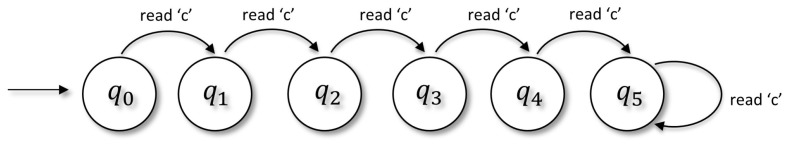
Overview of a state machine. The process reads the first character and, for every “c” found, a transition is realized.

A very simple example of a state machine is the one that models a character counting in a processed string. Let us suppose the counting program is fed by a string $\left\{a_{0},a_{1},\ldots ,a_{n}\right\}$ and count the number of matches of a specific character ‘*c*’ up to the limit value 5. The scheme is illustrated in Figure 11 and Table 1 lists the rules of the process.

$$\begin{array}{cc} States & :=\{q_{0},q_{1},q_{2},q_{3},q_{4}\}\\ Start\;state & :=q_{0}\\ Alphabet & :=\left\{a,b,\ldots z,A,B,\ldots ,Z\right\}\\ Transitions & :=\delta \left(q_{i},\prime c^{\prime }\right)\;\to \;q_{i+1|i<5}\\ Final\;states & :=\left\{q_{1},q_{2},q_{3},q_{4}\right\} \end{array}$$

In the above example, the finite automaton represents a process that consumes every character of a given string and will end counting $q_{n}\cdot n$ characters when $q_{n}$ is where it stopped or will find at least five matches if the final state is $q_{5}$.

As a tool for the proof of security of protocols, FSA can represent the behavior of players from a high-level viewpoint. Thus, the desired properties of the protocol are described as states, and the modeling process intends to enumerate all possible actions of the players as transitions among these states, pointing what are the (in)secure states, analyzing the probability of reaching each of them.

The typical approach to proofs of security using FSA is first to show that the protocol is correct for honest players. Then, dishonest players are added to the model, and the security is demonstrated by considering how these players can trouble the protocol. To develop the proof of security of our proposed protocol, we first show that the protocol is correct considering that honest players who know some secret key, by running the protocol, can reach the authenticated state, i.e., another player, the owner of the service, will be convinced about the identity of those players. After that, we demonstrate that it is infeasible to the adversary to reach that same state without the knowledge of that secret information, which will allow us to prove the security concerning the behavior of the honest players and the adversary.

### 7.2. The Invariant Principle

Proving the security of a protocol by modeling it as a finite state machine can be hard because this kind of proof must trace all the paths the protocol can take throughout the states. When there is a very high number of path possibilities, automatic tools are very useful for the proof. Another useful means is given by the *invariant principle* [70,71,72] that guides the proof by an induction method applied to finite state automata. An invariant is a statement that is proved to be true to all reachable states. In a proof by induction, we show that the invariant is true in the start state and demonstrate that it stays true to all reachable states.

An approach commonly taken for the proof of security using FSA is to show which states the adversary can reach, proving that among these states there are none where the invariant is violated [71]. For instance, if we suppose that the invariant is the adversary ignorance about a secret key, we can demonstrate the adversary never becomes authenticated; otherwise, if the adversary attains an authenticated state, this implies that the adversary breaks the supposed invariant. Furthermore, considering that, for a given state the invariant is true, even if the adversary increases his knowledge, regardless of what strategy he can use, this will not turn false the supposed invariant. More formally, this proof is defined as follows.

**Invariant**: An invariant is a predicate *P* over the states, such that whenever it is true for some state $q_{i}$, i.e., $P(q_{i})$ is true, and there is a transition $q_{i}\to q_{j}$, the predicate will also be true in state $q_{j}$, i.e., $P(q_{j})$ is true.

To get a security proof of our protocol, we follow the approach in [71], since in this work the authors expressed the behavior of honest and dishonest players as a finite state machine and formally define the security properties of the protocol regarding invariants.

### 7.3. Proving the Protocol Secure by Means of an FSA

This section describes the formalism used to model our protocol as an FSA, using the notation presented in [70]. Thus, hereafter, the work is devoted to modeling the behavior of the honest parties and the adversary through a finite state machine.

To this objective, we describe the operations performed by honest parties and the strategy of the adversary to break the protocol according to the approach in [70,71,72] which for our protocol is performed using the following script:

- Describe the entire protocol regarding honest players and adversary behavior, showing a list of states of the running protocol and the transition rules.
- Model the adversary behavior, considering what he can do to drive the protocol to an insecure state.
- Demonstrate the resilience of the protocol showing how the honest parties run to keep the invariants true, despite the dishonest participants, thus presenting the probability of these adversaries violating that invariant.

#### 7.3.1. The Protocol as a State Automata

We denote $q_{S},q_{R}$ the local states of the sender Alice and the receiver Bob, respectively. We translate all the network elements which can represent the network status to the local states of these parties. Thus, we include in the players states the messages $m_{i}$ to represent the current message in the local network of the *i*-th player. We also denote by $v_{i}$ all internal values, that is, all the current and past information the player knows, which includes all the information that he has seen in past executions of protocol. This way, the representation of the complete current state of the two parties is denoted respectively by:

(1) $$\left\{\begin{array}{cc} q_{S} & =<v_{S}^{i},m_{S}^{i}>,\\ q_{R} & =<v_{R}^{i},m_{R}^{i}>, \end{array}\right.$$

Furthermore, still following [70], we define the adversary state as $q_{A}=m_{A}^{1},m_{A}^{2},\ldots ,m_{A}^{M}$, the set of messages he has captured so far, added to the previous knowledge he keeps. The protocol’s global state is so denoted by the triple:

(2) $$\left[q_{S},\;q_{R},\;q_{A}\right],$$

In addition, we use the following formalism to denote the transitions rules:

(3) $$r_{k}^{\left(i|q_{A}\right)}=if\;C_{k}\left(q_{i}|q_{A}\right)\;then\;s\to s^{\prime },$$

The condition in the rule $C_{k}\left(s_{i}|q_{A}\right)$ depends on the current state $q_{i}=<v_{k}^{i},m_{k}^{i}>$, so the transition rule considers the current internal values the participant keeps, $v_{k}^{i}$, and what is the message intended to him $m_{k}^{i}$, ready to be read from the network. In the adversary case, this condition also depends on what he already knows $q_{A}$, so this is the general case presented above. Therefore, the specific case for the honest participants can be written as $r_{k}^{i}=if\;C_{k}\left(q_{i}\right)\;then\;s\to s^{\prime }$.

According to this rule, we give an example of a transition rule for the sender as follows:

(4) $$\begin{array}{cc} r_{S}^{i}=\mathrm{If}\;C_{S}\left(q_{S}^{i}=<v_{S}^{i},m_{S}^{i}>\right) & \;\mathrm{Then}\;\left[q_{S}^{i}=<v_{S}^{i},m_{s}^{i}>,q_{R}^{i}=<v_{R}^{i},\varepsilon >,\ldots \right]\\ & ⟶\left[q_{S}^{i+}=<v_{S}^{i+},\varepsilon >,q_{R}^{i+}=<v_{R}^{i},m_{R}^{i}>,\ldots \right] \end{array}$$

**Figure 12:**
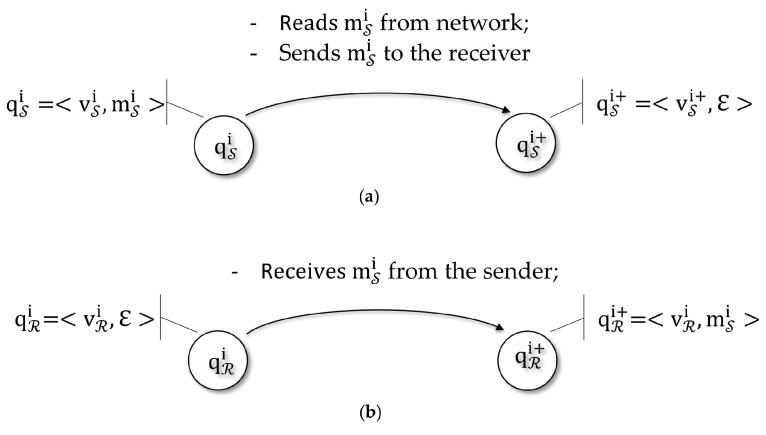
An overview of transition rules on a finite state machine: (**a**) the sender transmits a message to the receiver after reading $m_{S}^{i}$; and (**b**) the receiver responds to the sender after he gets the sender message.

The above example demonstrates the case for a sender who is currently in the state $q_{S}^{i}=<v_{S}^{i},m_{S}^{i}>$, that is, keeping its internal variable $v_{S}^{i}$ and reading the incoming message $m_{S}^{i}$. Once the sender processes this message, it reacts by sending a message $m_{R}^{i}$ to the receiver and moves to the state $q_{S}^{i+}=<v_{S}^{i+},\varepsilon >$, that is, he has changed the knowledge in his memory and is now waiting for the next message, which is indicated by an empty message ε in the network queue. Therefore, the rule, when processed, changes the global state of the protocol. The transition of states shows that the receiver, as a destination of the message sent, has his internal state changed from $q_{R}^{i}$ to $q_{R}^{i+}$, keeping the transmitted message on the network queue ready to be processed. This new state for the receiver will so be considered for the rule of its next transition. The finite state diagram shown in Figure 12 illustrates this dynamic.

#### 7.3.2. The FSA States for the Players

**Figure 13:**
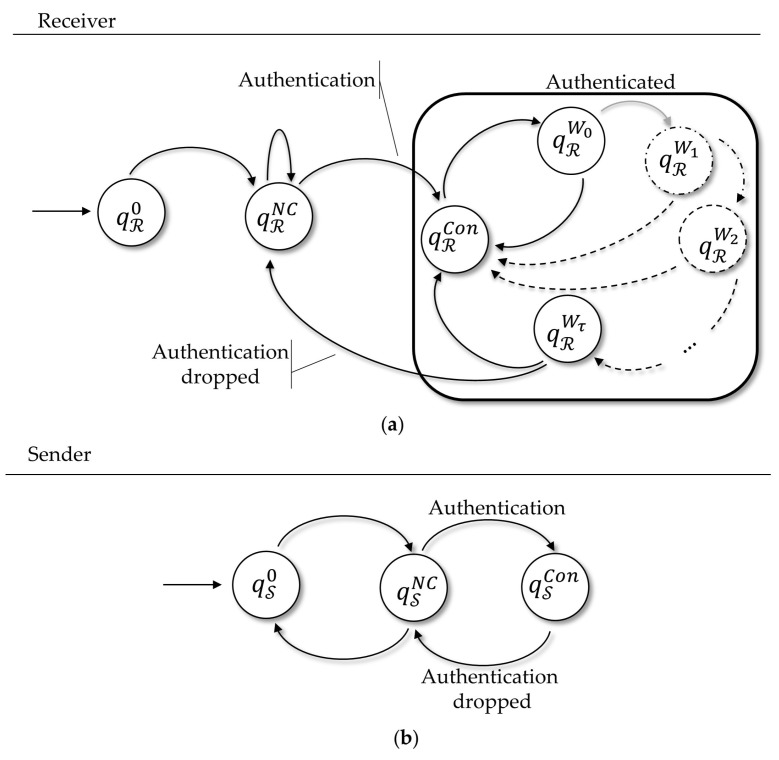
The finite state machine for the receiver (**a**) and the sender (**b**) in our proposed protocol.

Once the syntax is introduced, and referring to Figure 13, we present the description of our proposed protocol as the following set of states and transition rules:
**States for the receiver**$q_{R}^{0}$The initial state of the protocol, no transmission done yet;$q_{R}^{NC}$This represents the state of no authentication. If the sender has tried to send network communications to establish the authentication, it was not successful;$q_{R}^{Auth}$The receiver recognizes the authentication signal sent by the sender and keeps this information in his internals variables;$q_{R}^{W_{i}},i\geq 0$The receiver was waiting for a new authentication signal because there were *i* sequences of n packets with no valid signature;$q_{R}^{W_{\tau }}$Threshold limit was reached without the valid recognizable communications from the sender that could keep the authenticated status on.
**States for the sender**$q_{S}^{0}$The initial state of the protocol, no transmission done yet;$q_{S}^{NA}$This represents the state of no authentication awareness. If the sender has tried to send network communications to be identified, it was not successful;$q_{S}^{Auth}$Represents the states of being authenticated.
**Adversary states**$q_{A}^{0}$Initial state of the protocol, no transmission done yet;$q_{A}^{NA}$Represents the state of no authentication;$q_{A}^{Auth}$Represents the states where the adversary reaches a successful authentication.

#### 7.3.3. The Transition Rules for the Players

This section presents the transition rules for the participants of our proposal, writing them in the syntax introduced earlier.


**Receiver rules**

$r_{R}^{0}$
= $\mathrm{If}\;C_{R}(q_{R}^{0}=<v_{s}^{i},m_{S}^{0}>$
**Then**
$\left[\ldots ,q_{R}^{0},\ldots \right]⟶\left[\ldots ,q_{R}^{NA},\ldots \right]$
$r_{R}^{NA}$
= $\mathrm{If}\;C_{R}\left(q_{R}^{NA}=<v_{R}^{i},m_{S}^{i}>;Verify\left(m_{R}^{i}\right)\right)$
**Then**
$\left[\ldots ,q_{R}^{NA},\ldots \right]⟶\left[\ldots ,q_{R}^{Auth},\ldots \right]$
$r_{R}^{Auth}$
= $\mathrm{If}\;C_{R}\left(q_{R}^{Auth}=<v_{R}^{i},m_{R}^{i}>;\neg Verify\left(m_{R}^{i}\right)\right)$
**Then**
$\left[\ldots ,q_{R}^{Auth},\ldots \right]⟶\left[\ldots ,q_{R}^{W_{0}},\ldots \right]$
$r_{R}^{W_{j}}$
= $\mathrm{If}\;C_{R}\left(q_{R}^{W_{j}}=<v_{R}^{j},m_{R}^{j}>;Verify\left(m_{R}^{j}\right)\right)$
**Then**
$\left[\ldots ,q_{R}^{W_{j}},\ldots \right]⟶\left[\ldots ,q_{R}^{Auth},\ldots \right]$
$r_{R}^{W_{j}}$
= $\mathrm{If}\;C_{R}\left(q_{R}^{W_{j}}=<v_{R}^{j},m_{R}^{j}>;\neg Verify\left(m_{R}^{j}\right);j<\tau \right);$
**Then**
$\left[\ldots ,q_{R}^{W_{j}},\ldots \right]⟶\left[\ldots ,q_{R}^{W_{j+1}},\ldots \right]$
$r_{R}^{W_{\tau }}$
= $\mathrm{If}\;C_{R}\left(q_{R}^{W_{\tau }}=<v_{R}^{\tau },m_{R}^{\tau }>;\neg Verify\left(m_{R}^{\tau }\right)\right)$
**Then**
$\left[\ldots ,q_{R}^{W_{\tau }},\ldots \right]⟶\left[\ldots ,q_{R}^{NA},\ldots \right]$


**Sender rules**

$r_{S}^{0}$
= $\mathrm{If}\;C_{R}\left(q_{S}^{0}\right)$
**Then**
$\left[q_{S}^{0},\ldots \right]⟶\left[q_{S}^{NA},\ldots \right]$
$r_{S}^{NA}$
= $\mathrm{If}\;C_{R}\left(q_{R}^{NA}\right)$
**Then**
$\left[q_{S}^{NA},q_{R}^{NA}=<v_{R}^{NA},\varepsilon >,\ldots \right]⟶\left[q_{S}^{Auth},q_{R}^{NA+}=<v_{R}^{NA},m_{S}^{NA}:Sign>,\ldots \right]$
$r_{S}^{W_{i}}$
= $\mathrm{If}\;C_{R}\left(q_{R}^{W_{i}}\right)$
**Then**
$\left[q_{S}^{W_{i}},q_{R}^{W_{i}}=<v_{R}^{i},\varepsilon >,\ldots \right]⟶\left[q_{S}^{Auth},q_{R}^{W_{i}+}=<v_{R}^{i},m_{S}^{W_{i}}:Sign>,\ldots \right]$

#### 7.3.4. The Proposed Protocol Invariants

This section aims to demonstrate the security of the proposed protocol by defining its invariants and showing how they relate to the security properties we want to hold. This approach consists in proving that the invariants keep valid in face of attacks from the adversary.

***Invariant 1**: The adversary does not know the secret key for a valid signature*. This invariant gives us the certainty that whoever starts the protocol being unable to create a valid signature of a message will end the protocol still unable to sign messages, despite what this entity learns or sees. This also tells us that, if a dishonest player wants to get authenticated, he will not succeed since if it reaches the authenticated state it will break the invariant validity. The adversary, despite all the information he gets, will be unable to use it to synthesize signatures Synth(e); otherwise, it has earned the secret key. The honest sender capability of signature will be represented here by $Sign\left(m\right),\;m\in {\left\{0,1\right\}}^{*}$ function.

To demonstrate that this variant holds during all the reachable states, we will on the states which represent the authenticated status, i.e., $Q=\{q_{R}^{Auth},q_{R}^{W_{0}}\ldots ,q_{R}^{W_{\tau }}\}$. It is enough to demonstrate that to reach any of these states a sender must make the receiver transit from the state $q_{R}^{NA}$ to the state $q_{R}^{Auth}$. Therefore, an honest participant will reach that by using the rule:

(5) $$\begin{array}{cc} r_{S}^{NA}=\mathrm{If}\;C_{S}\left(q_{R}^{NA}\right) & \;\mathrm{Then}\left[q_{S}^{NA},q_{R}^{NA}=<v_{R}^{i},\varepsilon >,\ldots \right]\\ & ⟶\left[q_{S}^{Auth},q_{R}^{NA+}=<v_{R}^{i},m_{R}^{NA}:Sign>,\ldots \right] \end{array}$$

That is, the honest sender uses a valid key to create a valid signature of a message and append it to the message $m_{R}^{NA}$ and sends this message to the receiver.

If the adversary can create a valid signature and therefore reach any of the states in $Q=\{q_{R}^{Auth},q_{R}^{W_{0}}\ldots ,q_{R}^{W_{\tau }}\}$, then he violates the invariant. Thus, he would break the invariant because the rule that makes the adversary convince his authenticity to the receiver is the one that uses all the collected information $q_{A}$ as support for the adversary to synthesize a signature $Synth(q_{A})$:

(6) $$\begin{array}{cc} r_{S}^{NA}=\mathrm{If}\;C_{S}\left(q_{S}^{NA}|q_{A}\right) & \;\mathrm{Then}\left[q_{S}^{NA},q_{R}^{NA}=<v_{R}^{i},\varepsilon >,\ldots \right]\\ & ⟶\left[q_{S}^{Auth},q_{R}^{NA+}=<v_{R}^{i},m_{S}^{NA}:Synth\left(q_{A}\right)>,\ldots \right] \end{array}$$

However, we already know that doing it with success will be a violation of a fundamental property of the considered digital signature scheme: infeasibility to forge a valid signature. In addition, we reiterate that it cannot be done despite the previous knowledge $q_{A}$ acquired by the adversary.

Furthermore, let us suppose the adversary has convinced the receiver about his identity and led the receiver to some of the states in *Q* and let us also assume the invariant was not violated, that is, the adversary still does not know the valid key to sign messages but became able to do it using some other strategy to synthesize a signature that the receiver recognizes as a valid one. In this case, the adversary will be able to stay in the set of states *Q* the same number of times he can synthesize new signatures as such. This is because the rule that makes the receiver to change the state from any of the waiting states $\left\{q_{R}^{W_{i}}\right\}$ to the $state{,q}_{R}^{Auth}$ requires a (synthesized) valid signature. Otherwise, the sender knows the secret key, which violates the considered invariant.

***Invariant 2**: An authenticated sender stays in the authenticated state*. This invariant must hold if the protocol is resilient to adversary attacks to thwart the authentication mechanism, e.g., when a malicious adversary controlling the network has interfered in messages needed to establish the authentication relation.

It is worth observing that a packet network flow and congestion control mechanism will possibly try to retransmit any packet dropped by an adversary. However, in our proof, we do not have any supposition about the flow and congestion control of the underlying network protocols. Thus, let us suppose the adversary has successfully stolen packets in a way the receiver is not aware. At the same time, we assume that the adversary can use a stolen packet in the future and fit it in a new network flow. In addition, the adversary can drop the signature from the packet and resend the packet just with the plaintext. Here, we do not make a distinction between these two cases and consider as the same effect if the adversary drops packet signatures or the whole packets.

The proof of this invariant can be given modeling the involved protocol states as a Markov chain, where the whole protocol dynamic described by the presented finite state machine will be represented by a transition matrix *P* as expressed in Equation (7). We denote δ the probability of the adversary interfering in all valid signed packets, despite unknowing which are the valid ones. Thus, if the adversary wins targeting all the valid signed packets, we represent his strategy by the probability δ=1, while if he fails we have δ=0. In the next subsection, we give a measure of how δ depends on the sender signing strategy.

(7) $$P=\left[\begin{array}{ccccccc} & Auth & W_{0} & W_{1} & \cdots & W_{\tau } & NA\\ Auth & 1-\delta & \delta & 0 & \cdots & 0 & 0\\ W_{0} & 1-\delta & 0 & \delta & \cdots & 0 & 0\\ W_{1} & 1-\delta & 0 & 0 & \cdots & 0 & 0\\ \vdots & \vdots & \vdots & \vdots & \ddots & \vdots & \vdots \\ W_{\tau } & 1-\delta & 0 & 0 & \cdots & 0 & \delta \\ NA & 1-\delta & 0 & 0 & \cdots & 0 & \delta \end{array}\right]$$

In the matrix presented in Equation (7), the elements Auth, $W_{0},\ldots ,\;W_{\tau },\;NA$ correspond to the index of states in the finite automata shown in Figure 13 and each of the matrix cells $P_{m,n}$ is the probability of transition between the correspondent states in the index. Based on this matrix, we analyze the stationary distribution of the Markov chain, since it is well known that the stationary distribution tells us the proportion of how many times the process stays in each state in a long time run of the Markov process. Thus, we infer from this distribution what is the chance of a sender losing the authenticated status, considering the interference that the adversary can make by controlling the network channel.

This way, we consider the Markov process with the state space $S=\{q_{R}^{Auth},q_{R}^{W_{0}}\ldots ,q_{R}^{W_{\tau }},q_{R}^{NA}\}$ and the matrix of transition probabilities P. Furthermore, if $\pi_{j},\;j\in S$ is a distribution over S. That is, π is a row vector with |S| elements such that $\sum_{j}\pi_{j}=1$, $\pi_{j}\geq 0$, for every j∈S. Then, if some initial distribution $\pi_{0}$ makes the Markov chain stationary with stationary distribution π, we have

(8) $$\pi_{j}=\pi \cdot P$$

Then, for all $j\in S,\pi_{j}$ is the dot product between π and the *j*th column of *P*:

(9) $$\pi_{j}=\sum_{i\in S}\pi_{i}\cdot P_{i,j}$$

Now we find the distribution of the stationary state for the Markov chain modeling our protocol. From matrix *P* we have:

(10) $$1=\pi_{Auth}+\pi_{W_{0}}{+\;\pi }_{W_{1}}+\ldots +\pi_{W_{\tau }}{+\;\pi }_{W_{NA}}$$

(11) $$\pi_{Auth}=\left(1-\delta \right){\cdot \pi }_{Auth}+\left(1-\delta \right){\cdot \pi }_{0}+\ldots +\left(1-\delta \right)\cdot \pi_{\tau }+\left(1-\delta \right){\cdot \pi }_{NA}$$

(12) $$\pi_{W_{0}}=\delta .\pi_{Auth}$$

(13) $$\pi_{W_{1}}=\delta \cdot \pi_{W_{0}}=\delta^{2}\cdot \pi_{Auth}$$

(14) $$\pi_{W_{2}}=\delta \cdot \pi_{W_{1}}=\delta^{3}\cdot \pi_{Auth}$$

⋮

(15) $$\pi_{W_{\tau }}=\delta \cdot \pi_{W_{\tau -1}}=\delta^{\tau +1}\cdot \pi_{Auth}$$

(16) $$\pi_{NA}=\delta \cdot \pi_{W_{\tau }}+\delta \cdot \pi_{NA}=\delta^{\tau +2}\cdot \pi_{Auth}+\delta \cdot \pi_{NA}$$

Doing substitutions in (10), we reach:

(17) $$\left\{\begin{array}{cc} 1 & =\pi_{Auth}+\pi_{W_{0}}+\pi_{W_{1}}+\ldots +\pi_{W_{\tau }}+\pi_{W_{NA}}\\ & =\pi_{Auth}+\delta \cdot \pi_{Auth}+\delta^{2}\cdot \pi_{Auth}+\ldots +\delta^{\tau +2}\cdot \pi_{Auth}+\delta \cdot \pi_{NA}\\ & =\pi_{Auth}\cdot \left(1+\delta +\delta^{2}+\ldots +\delta^{\tau +2}\right)+\delta \cdot \pi_{NA} \end{array}\right.$$

Using the general form for a sum of a geometric series and doing substitutions, we reach:

(18) $$\left\{\begin{array}{cc} \pi_{NA} & =\frac{\delta^{\left(\tau +2\right)}}{1-\delta }\cdot \pi_{Auth}\\ 1 & =\pi_{Auth}\cdot \frac{1-\delta^{\left(\tau +3\right)}}{1-\delta }\cdot \delta \cdot \pi_{NA}\\ \pi_{Auth} & =\frac{1-\delta }{1+\delta^{\tau +2}-\delta^{\tau +3}} \end{array}\right.$$

Finally, we use $\pi_{Auth}$ to determine how much the adversary can thwart an authentication relation:

(19) $$\pi_{Auth}=\frac{\delta^{\tau +2}}{1+\delta^{\tau +2}-\delta^{\tau +3}}$$

Observing this indicator, we can see that when the adversary does not interfere with the packets, whether because he could not catch the valid signature or whatever else, the probability of the sender staying in the state $\pi_{Auth}$ will be driven just by the sender behavior. Otherwise, when the malicious participants try to interfere in the signed messages, thwarting all the valid ones with probability δ, the higher is δ, the lower is the chance for the process to stay in $\pi_{Auth}$, hence δ=1 implies $\pi_{Auth}=0$.

#### 7.3.5. How δ Depends on the Sender Signing Mechanism

If the adversary produces a low interference rate δ≪1, the process will stay on the authenticated states with higher probability. We argue that no effective adversary strategy gives him a good chance of thwarting the authentication of a sender, except if the adversary drops most of the packets or targets the valid signed ones because he has broken the signature secrets.

As a numerical example, let us suppose a scenario in which the following parameters are defined: one round will be executed for every n=100 packets and we produce and process a signature for every Σ=10 of these packets, signing with a designated and valid key just one of them, σ=1. The receiver is set to tolerate up to τ=3 of these rounds with no valid signature.

If the adversary, for example, tries to drop just one packet, this gives a rate of δ=1/10 which will result in $\pi_{NA}$ very close to zero (9.9 × 10${}^{-6}$), i.e., the chance is of 99,999% for the receiver staying in one of the states $q_{R}^{Auth},q_{R}^{W_{0}},\ldots ,q_{R}^{W_{\tau }}$.

**Figure 14:**
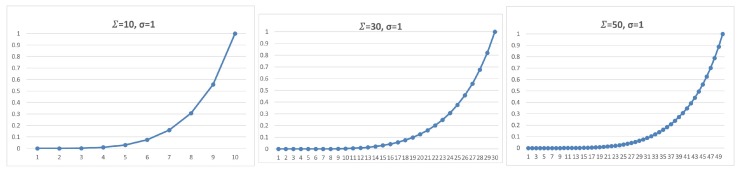
Relation between the number of packets the adversary manipulates and the probability $\pi_{NA}$ of the receiver not holding the authenticated status for a sender.

By extending this example, it is possible to present numerical results calculated for the proposed protocol performance. We consider the same setup in which the round is executed for every n=100 packets and just one packet is signed with a designated and valid key, so σ=1. The receiver still tolerates up to τ=3 of these rounds with no valid signature. We plot in Figure 14 the results for the values of Σ=10, Σ=30 and Σ=50, showing the probability of the receiver not holding the authentication status of a sender when the adversary drops a number of packets varying from 1 up to the total Σ, 10, 30 and 50, respectively.

Furthermore, the case where the sender puts more than one valid signature must be analyzed. The sender can fit the σ>1 valid signatures in Σσ ways, which increases the hardness for an adversary to interfere with the scheme, mainly because it is needed to hit at least σ valid signatures. Thus, the adversary attacking up to σ−1 of them will not interfere in the scheme. The fact that the proposed protocol mixes valid and non-valid signatures amplifies the hardness of the attack. For instance, when we combine Σ=10 and σ=2, we have 102=42 options on how we can fit the signatures. Thus, while σ=1 required the attacker to manipulate only five packets to get a 50% chance to hit the valid signed message, with σ=2 the attacker will need to interfere in at least seven packets to get the same probability of successfully hitting the designated signed packets.

**Figure 15:**
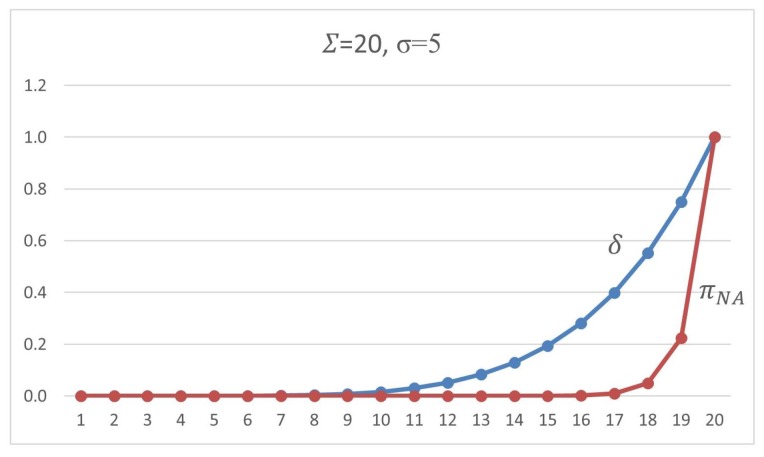
Relation between the number of packets the adversary manipulates, the probability of hitting valid signatures δ and the probability $\pi_{NA}$ of invalidating an authentication, working with σ=2. In this case, the adversary gets a higher chance only after dropping most of the packets (more than 19 packets in 20).

Another numerical example is presented considering Σ=20 and σ=5, n=1000. In this case, the sender has 205=15,504 different ways to dispose of the valid/non-valid signed packets. Figure 15 illustrates the chances when the adversary attacks a number of packets varying from 1 to 20 packets. The figure shows that the adversary by interfering in less than five packets will get no chance to thwart an authentication.

That said, the reasoning proves that the Markov process representing our proposed protocol authentication mechanism has a stationary state that shows the protocol as resilient in face of adversary attacks. Whenever the sender wants to establish an authentication relation with the receiver, he will be identified with success. Otherwise, there would be a violation of the security assumption on the protocol setup.

The resilience gain resulting from using more than one signed message and mixing it among the others can be amplified when we choose a combination of σ among Σ in its maximum, i.e., when σ≅σ/2. Thus, the adversary has still fewer chances when he tries a guess of d<Σ. When he chooses d=Σ−1, the chances of matching are easily verified as:

(20) ΣσΣ−1σ≅2, when σ≅Σ/2

**Figure 16:**
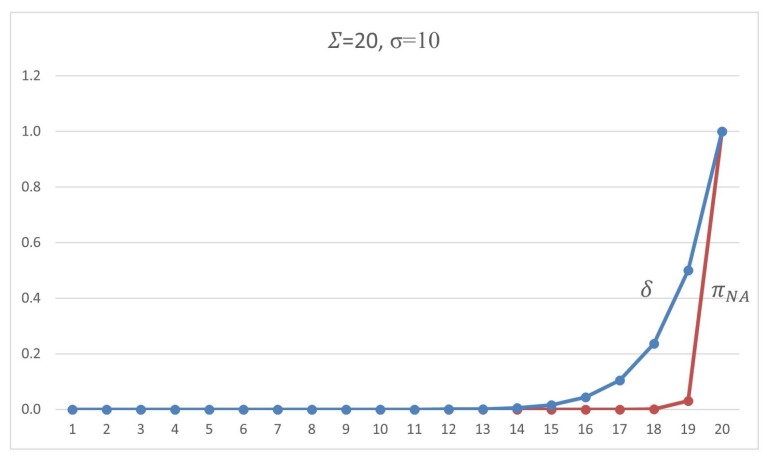
Relation between the number of packets the adversary manipulates, the probability of hitting valid signatures δ and the probability $\pi_{NA}$ of invalidating an authentication, working with σ=10. In this case, the adversary has a chance only after dropping the total of the packets. With these settings, the all-or-nothing nature of our defense scheme is clear.

Hence, Equation (20) tells us that if the adversary does not try an all-guess interference in the signed packets, even if he does not proceed for all but one packet, the chance δ of matching the signed packets will be dropped by 1/2. This ability to hide the valid signatures will have a huge effect that can be verified in Figure 16.

## 8. Conclusions

We have presented a protocol that allows authentication between a sender and a receiver in a context of packet-switching networks. Our solution is innovative in the way we apply digital signatures, and therefore our contribution comes from the original way we use cryptography for building an anti-DoS defense mechanism which executes a strategy of packet authentication-based traffic filtering to counter DoS attacks. Our proposal does not rely on an expensive public-key infrastructure and requires light cryptography machinery that is suitable in the context of the Internet of Things.

We also presented how our solution is resistant to known denial of services attacks. That was done using the formal specification of the proposed protocol, describing it through the finite state formalism, and thus we have formally demonstrated the security of the proposed scheme.

Experimental validation of our proposal in a controlled testing environment is planned. Indeed, implementation details of any security protocol involve secure software development processes and the analysis of trust assumptions that are out of the scope of the present paper, being appropriate for further study. An experimental evaluation presents new challenges that we consider, for instance, the choice of a signature algorithm to be used, since there are recent proposals of short and efficient algorithms for designated signatures. We must also choose the security parameters and the network parameters, among other questions such as language and simulation environment.

## Figures and Tables

**Table 1:** The relation among the current state, the symbol being read, and the after state.

Old State	Symbol	New State
$q_{0}$	‘c’	$q_{1}$
$q_{1}$	‘c’	$q_{2}$
$q_{2}$	‘c’	$q_{3}$
$q_{3}$	‘c’	$q_{4}$
$q_{4}$	‘c’	$q_{5}$
$q_{5}$	‘c’	$q_{5}$
